# Optogenetic control of the Bicoid morphogen reveals fast and slow modes of gap gene regulation

**DOI:** 10.1016/j.celrep.2022.110543

**Published:** 2022-03-22

**Authors:** Anand P. Singh, Ping Wu, Sergey Ryabichko, João Raimundo, Michael Swan, Eric Wieschaus, Thomas Gregor, Jared E. Toettcher

**Affiliations:** 1 Lewis Sigler Institute for Integrative Genomics, Princeton University, Princeton, NJ 08544, USA; 2 Department of Molecular Biology, Princeton University, Princeton, NJ 08544, USA; 3 Department of Physics, Princeton University, Princeton, NJ 08544, USA; 4 These authors contributed equally; 5 Lead contact

## Abstract

Developmental patterning networks are regulated by multiple inputs and feedback connections that rapidly reshape gene expression, limiting the information that can be gained solely from slow genetic perturbations. Here we show that fast optogenetic stimuli, real-time transcriptional reporters, and a simplified genetic background can be combined to reveal the kinetics of gene expression downstream of a developmental transcription factor *in vivo*. We engineer light-controlled versions of the Bicoid transcription factor and study their effects on downstream gap genes in embryos. Our results recapitulate known relationships, including rapid Bicoid-dependent transcription of *giant* and *hunchback* and delayed repression of *Krüppel*. In addition, we find that the posterior pattern of *knirps* exhibits a quick but inverted response to Bicoid perturbation, suggesting a noncanonical role for Bicoid in directly suppressing *knirps* transcription. Acute modulation of transcription factor concentration while recording output gene activity represents a powerful approach for studying developmental gene networks *in vivo*.

## In brief

Singh et al. investigate how rapid changes in nuclear Bicoid levels dynamically regulate expression of its target genes. Combining optogenetic stimulation and a simplified genetic background, their results recapitulate the canonical role of Bicoid as a transcriptional activator and reveal a possible noncanonical role in direct *kni* repression.

## Graphical Abstract

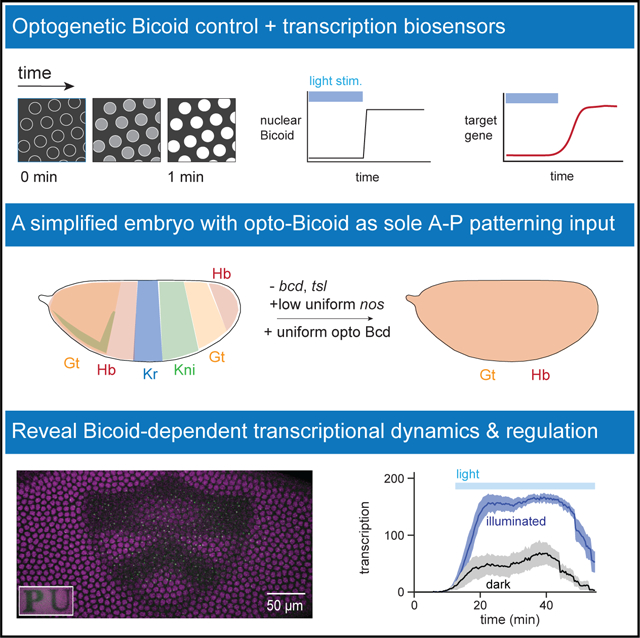

## INTRODUCTION

Gene networks play a crucial role in developmental patterning, transforming rudimentary positional cues into a multitude of sharply defined domains of gene expression. Such networks are typically characterized by redundant inputs, to ensure that gene expression is initialized appropriately, and feedback connections between genes in the network, to ensure a consistent patterning response. Information from these inputs is integrated at enhancers that bind multiple transcription factors and may control gene expression through transient interactions with promoters or longer-term alterations in chromatin structure and accessibility. Understanding how networks function requires knowing the time scales over which individual components operate.

**Figure 1.**
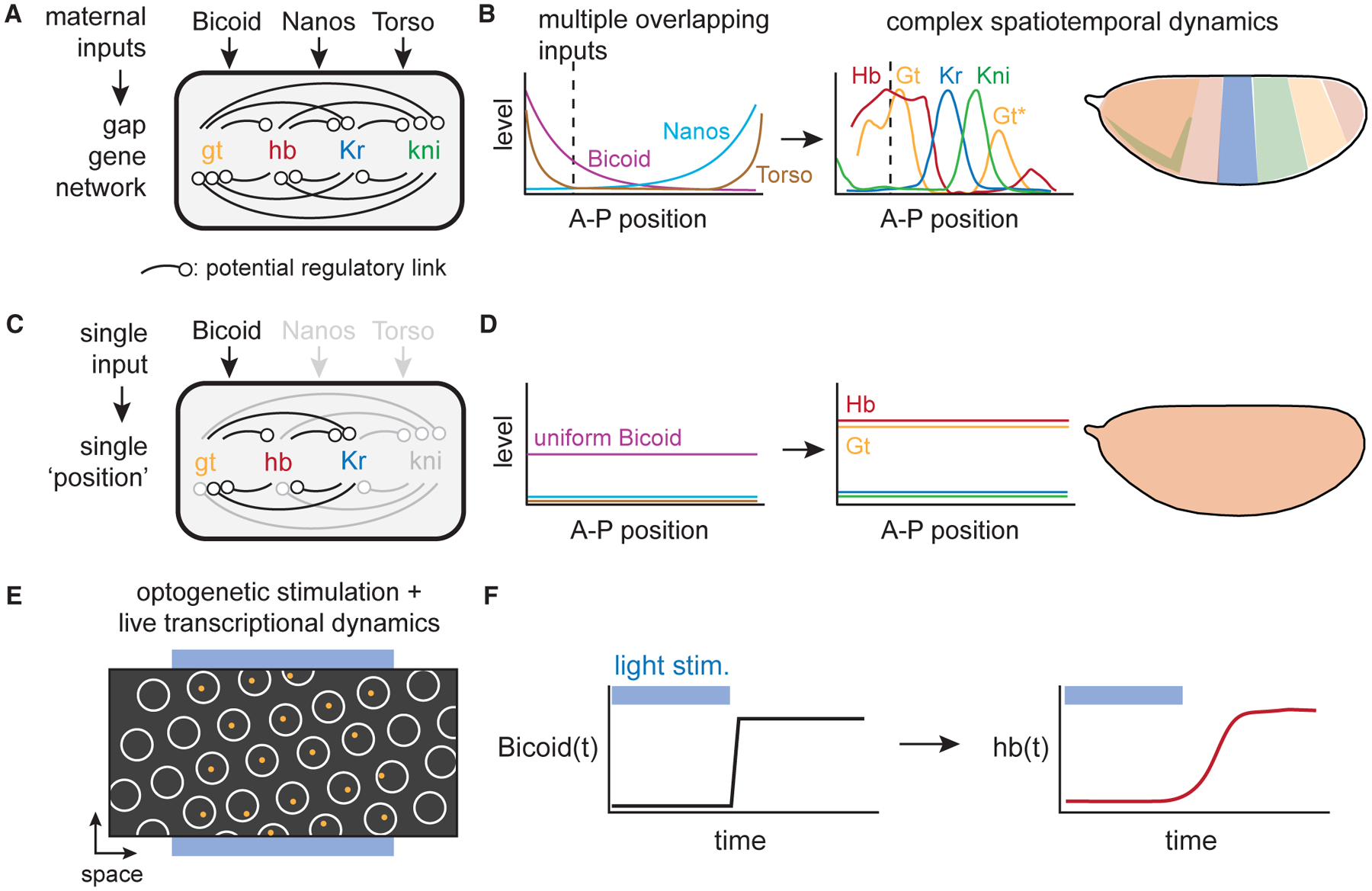
Studying Bicoid-dependent gap gene responses using a stimulus-response approach in single-input embryos (A and B) The endogenous gap gene network depends on three maternally supplied inputs (Bicoid, Nanos, and Torso) and many potential feedback and crosstalk links (in [A]) to generate bands of gap gene expression across the embryo (in [B]). A detailed understanding of this network is made challenging by the presence of multiple redundant inputs and complex dynamics as spatial patterns shift over time. (C and D) To study the effects of the Bicoid transcription factor on gap gene expression, we set out to construct a reduced-complexity network where Bicoid is the sole maternally supplied input to the network (in [C]) and spatial patterning is eliminated (in [D]). (E and F) To define the strength, duration, and dynamics of Bicoid-dependent gap gene responses, we acutely perturb nuclear Bicoid levels using an optogenetic technique and monitor resulting gene expression in individual nuclei using live transcription reporters. The y axis on the left in (F) shows nuclear Bicoid concentration varying across time and on the right shows transcription rate for one representative Bicoid target gene, *hb*.

The gap gene network of the early *Drosophila* embryo is a canonical example of such a sophisticated pattern-forming system. In this network, the expression of four core transcription factors—the gap genes *giant* (*gt*), *hunchback* (*hb*), *Krüppel* (*Kr*), and *knirps* (*kni*)—is initiated by three partially redundant sources of positional information that are maternally deposited in the egg. These maternally supplied inputs include an anterior-to-posterior gradient of the Bicoid (Bcd) transcription factor, a posterior-to-anterior gradient of the Nanos RNA binding protein, and Torso receptor tyrosine kinase signaling at the anterior and posterior poles. In addition to responding to maternal inputs, the four gap genes further regulate themselves and one another to generate bands of gene expression that are essential for specifying the body plan (Figures 1A and 1B) (Briscoe and Small, 2015; Jaeger, 2011).

A useful first step in disentangling such networks has been to characterize transcriptional responses under conditions where input information has been reduced to single components or flattened so that all cells in the embryo see the same input values (Figures 1C and 1D). For example, when all anterior-posterior (A-P) patterning inputs except Bcd are eliminated, the pattern is reduced and shifted relative to wild type, but the fundamental sequence of all four gap genes is maintained (Petkova et al., 2019). Similarly, flattening spatial patterns generates embryos that reflect a single A-P position along the wild-type gradient without the complexity of network components diffusing across shifting gene expression boundaries (Hannon et al., 2017; Ing-Simmons et al., 2021; Jaeger et al., 2004b). While such simplified systems can provide useful insights about Bcd-dependent features of the network, they do not distinguish between direct and indirect effects or long- and short-term mechanisms.

Real-time measurement of responses to acute perturbations provides a powerful approach to define the individual links in a complex gene network (Rullan et al., 2018; Wilson et al., 2017), revealing the sign, magnitude, and timescale with which a transcription factor input affects expression of specific target genes. Differences in response kinetics can also distinguish direct interactions (e.g., where a transcription factor directly regulates its target’s expression) from indirect links (where an intermediate gene product must first be synthesized), which can be crucial when the input influences a downstream gene through multiple regulatory paths. Although such rapid stimulus-response experiments have been traditionally difficult to perform *in vivo*, the recent advent of optogenetic perturbations and live biosensors of gene expression offers the possibility of defining transcriptional input/output relationships with unprecedented resolution (Figures 1E and 1F) (de Mena et al., 2018; Farahani et al., 2021; McFann et al., 2021; Patel et al., 2019; Rullan et al., 2018).

Here, we set out to use the fly embryo as a laboratory for dissecting how the Bcd transcription factor controls position-specific target gene expression, using fast stimulus-response measurements in simplified embryos that lack redundant inputs and spatial patterns. We generated a series of light-sensitive Bcd constructs whose nuclear-cytosolic localization can be shifted in less than a minute using blue light. We introduced these constructs in embryos that lack all other sources of A-P asymmetry, eliminating the protein gradients and shifting spatial distributions that typically complicate the study of patterning gene networks. When expressing optogenetic Bcd constructs with different activity levels, these synthetic, spatially homogeneous embryos mimic either anterior, central, or posterior embryonic positions, offering a toolbox for studying the real-time transcriptional responses to acute perturbation of a developmental patterning cue. Combining acute optogenetic Bcd perturbation with live-embryo biosensors of gap gene expression reveals both rapid and delayed modes of Bcd-dependent regulation. Anteriorly expressed gap genes *gt* and *hb* respond within minutes to changes in Bcd concentration, consistent with a direct role for Bcd in their transcriptional activation. In contrast, the medial gap gene *Kr* exhibits a delayed and inverted response, indicative of indirect Bcd-induced repression through an intermediate node. Finally, we report that the posteriorly expressed gap gene *kni* is transcribed within 3 min upon acute loss of nuclear Bcd, an unexpected response suggesting that Bcd acts directly to repress *kni* transcription without requiring new gene synthesis. Our approach, combining rapid nuclear-cytosolic shuttling of a transcription factor with real-time transcription measurements in a developmental gene-regulatory network, offers the possibility of dissecting regulatory links with unprecedented precision.

## RESULTS

### An activity series of optogenetic Bicoid constructs with rapid stimulus-response kinetics

**Figure 2.**
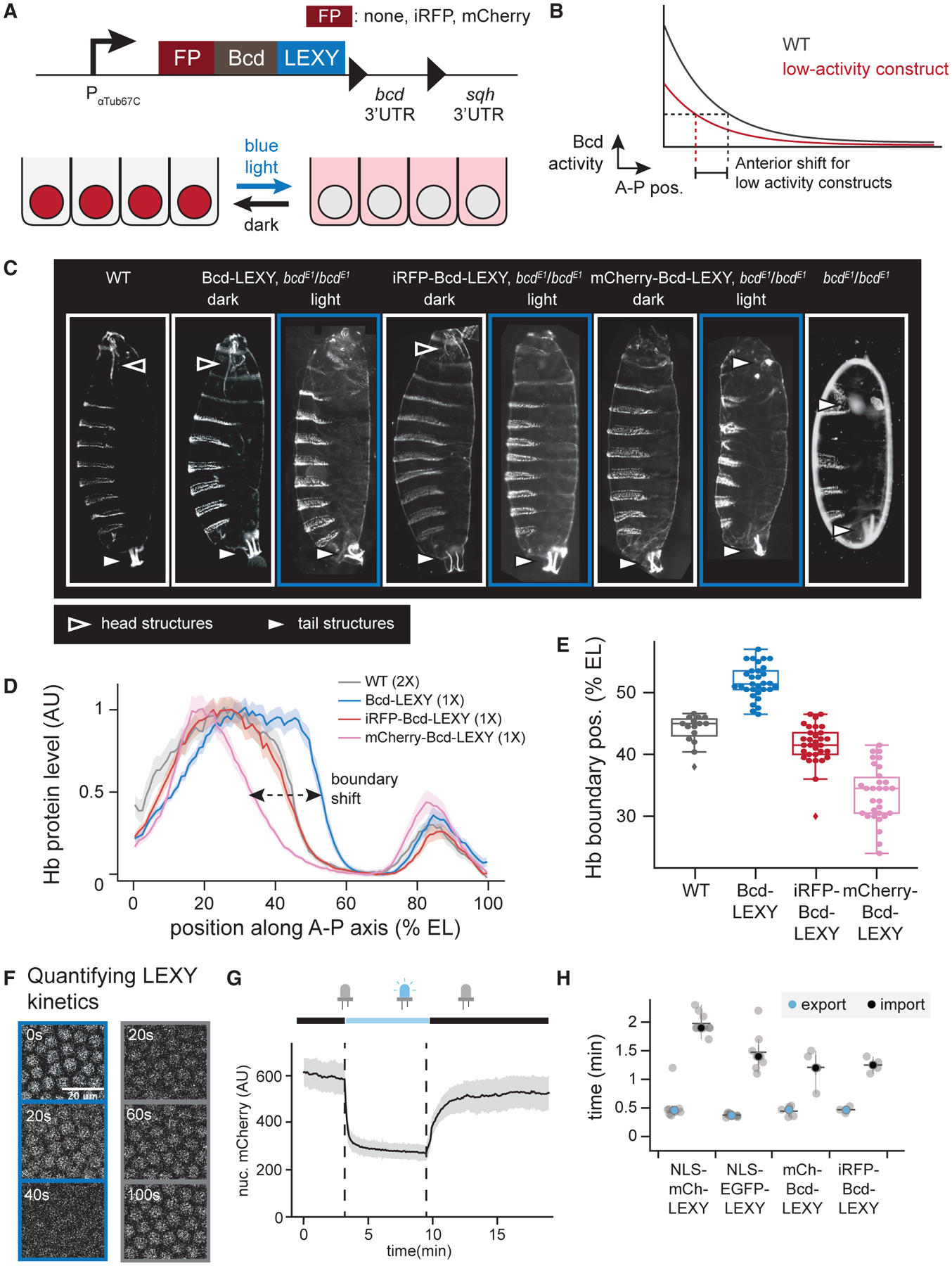
A series of optogenetic Bicoid constructs with variable activity and rapid kinetics (A) Bicoid was fused to various N-terminal fluorescent proteins and the LEXY optogenetic system at the C terminus. Light illumination at 450 nm exposes LEXY’s nuclear export sequence (NES), leading to an expected decrease in Bicoid transcriptional activity. (B) Bicoid constructs harboring weaker activity are expected to exhibit loss of anterior structures and an anterior-ward shift of gene expression patterns. (C) Larval cuticles for different Bcd-LEXY constructs under dark and light conditions. Anterior head and posterior tail structures are indicated with the outlined and shaded arrows. Illuminated embryos exhibit loss of anterior structures or duplication of posterior structures, indicating progressive loss of Bicoid activity. (D and E) Immunofluorescence for Hunchback (Hb) protein for three Bcd-LEXY constructs, compared with wild-type (WT) embryos. Embryos were collected and fixed under dark conditions. Hb levels were quantified as a function of position and genetic background in (D), with the boundary of anterior Hb expression quantified for individual embryos in (E). Bcd-LEXY exhibits high activity, whereas iRFP-Bcd-LEXY and mCherry-Bcd-LEXY exhibit progressively weaker activity as determined by the boundary of anterior Hb expression. Error bars show SEM in (D), boxes and whiskers represent 25^th^ and 75^th^ percentiles, minima and maxima, respectively, in (E). n = 15 for WT, n = 30 for the other three genotypes. (F and G) mCherry-Bcd-LEXY time course during optogenetic activation and deactivation. Representative images are shown in (F). Left: 0, 20, and 40 s after blue light was applied. Right: 20, 60, and 100 s after blue light was removed; quantification is shown in (G), with mean ± SD shown, averaged over >300 nuclei. (H) Quantification of import and export kinetics for four LEXY constructs; similar kinetics of translocation are observed for fluorescent Bicoid constructs and non-Bicoid-containing LEXY constructs. n = 9, 8, 4, and 4, respectively, for four LEXY constructs. Colored dots and error bars represent mean ± SD. See also Figure S1 and Table S1.

We engineered optogenetic constructs of Bcd to serve as rapidly switchable inputs to each gap gene. We fused Bcd to LEXY, an optogenetic tool based on the AsLOV2 protein domain whose nuclear export is reversibly triggered by blue light (Chen et al., 2020; Dowbaj et al., 2021; Kogler et al., 2021; Niopek et al., 2016; Viswanathan et al., 2021). Blue light illumination uncages a buried nuclear export sequence (NES) in LEXY’s C-terminal Jα helix; in the dark, NES activity is lost and Bcd’s noncanonical nuclear localization signal (NLS) (Grimm and Wieschaus, 2010) returns the fusion protein into the nucleus (Figure 2A). LEXY-based translocation typically produces a 5-fold change in nuclear protein concentration (Kogler et al., 2021; Niopek et al., 2016), whereas the natural Bcd gradient is thought to affect gene expression over a larger range, suggesting that Bcd constructs at various expression or activity levels may be required to probe gap gene responses at different embryonic positions. We thus tested a series of Bcd-LEXY constructs that were left either untagged or N-terminally fused to different fluorescent proteins, a modification previously observed to generate distinct Bcd activity levels (see STAR Methods) (Grimm and Wieschaus, 2010).

To assess the function of each Bcd-LEXY construct, we generated embryos harboring a single dosage as the sole Bcd source and assessed its function in the light and dark. Bcd is normally expressed in an anterior-to-posterior gradient, so conditions in which Bcd activity is reduced should lead to loss of anterior structures and/or an anterior shift of gap gene expression patterns (Figure 2B). Bcd-LEXY and iRFP-Bcd-LEXY embryos exhibited body segmentation and cephalic furrow position consistent with high Bcd activity in the dark, and embryos harboring a single copy of either allele hatched at rates of 70% and 42%, respectively (Figure 2B; Table S1). Blue light led to an apparent reduction in Bcd activity in Bcd-LEXY and iRFPBcd-LEXY embryos, characterized by the loss of mouth parts and thoracic segments at the anterior and a loss of embryo viability. mCherry-Bcd-LEXY-carrying embryos displayed weaker overall Bcd activity, as these embryos failed to form anterior structures in the dark and phenocopied *bcd*^*E1*^ loss-of-function embryos under illumination (Figure 2C, right).

We also measured expression of the canonical Bcd target gene Hunchback (Hb) in embryos harboring each of the three Bcd-LEXY constructs (Figures 2D and 2E). Compared with wild-type embryos, the position of the Hb boundary is shifted toward the posterior in Bcd-LEXY embryos and progressively shifts toward the anterior in iRFP-Bcd-LEXY and mCherry-Bcd-LEXY embryos, confirming that these three constructs form an activity series: Bcd-LEXY > iRFP-Bcd-LEXY > mCherry-Bcd-LEXY. A prior study reported Hb boundary positions as the gene dosage of Bcd was varied (Liu et al., 2013); comparing with these positions suggests that Bcd-LEXY represents Bcd activity at an equivalent dose of ~23× wild-type Bcd, iRFP-Bcd-LEXY is roughly equivalent to wild-type Bcd, and mCherry-Bcd-LEXY produces a dose of 0.53× wild-type Bcd. Immunostaining for Bcd revealed that these activity differences are partially explained by differences in expression level of the constructs (Figure S1A). Nevertheless, all three constructs were expressed at higher levels than wild-type Bcd, suggesting that fusion to LEXY and/or a fluorescent protein also leads to an apparent reduction in Bcd activity (Grimm and Wieschaus, 2010; Liu et al., 2013).

To assess nuclear-cytosolic translocation efficiency of our fluorescent protein (FP)-Bcd-LEXY constructs, we quantitatively characterized nuclear import and export dynamics for each construct as well as two LEXY-tagged fluorescent proteins that lacked any transcription factor fusions (NLS-mCherry-LEXY and NLS-EGFP-LEXY) (Figures 2F–2H; see STAR Methods for imaging details). Switching a 450 nm light on or off led to a rapid redistribution of each Bcd-LEXY construct in nuclear cycle 14 (NC14) embryos (e.g., mCherry-Bcd-LEXY in Figure 2G). Comparable dynamic responses were observed across all constructs, with light-induced nuclear export in 30 s and darkness-induced import in 1–2 min (Figure 2H). Illumination also produced nuclear export of similar magnitude and spatial precision, with a 4-fold change in nuclear concentration between dark and light conditions and a spatial precision of ~10–12 μm (one to two cells) (Figures S1B–S1D). These data establish the LEXY system as a tool for rapid and reversible modulation of nuclear transcription factor concentration during pre-gastrulation *Drosophila* embryogenesis. During preparation of this article, a LEXY variant harboring point mutations that slow down reversion to the dark state after photoactivation (Kawano et al., 2013) was reported for use in *Drosophila* embryos (Kogler et al., 2021). Our approach is also distinct from a prior optogenetic Bcd construct, where fusion of Bcd to the Cry2 oligomerization domain led to a potent, light-inducible dominant-negative response (Huang et al., 2017). While both prior optogenetic approaches can exhibit a wide overall dynamic range, they come at the cost of slower reversion to the active state upon a shift to darkness, and thus would be ill suited to acutely increasing nuclear Bcd levels to study rapid effects on transcription, a central goal of the current study.

### A reduced-complexity embryo for dissecting Bcd regulation of gap gene expression

**Figure 3.**
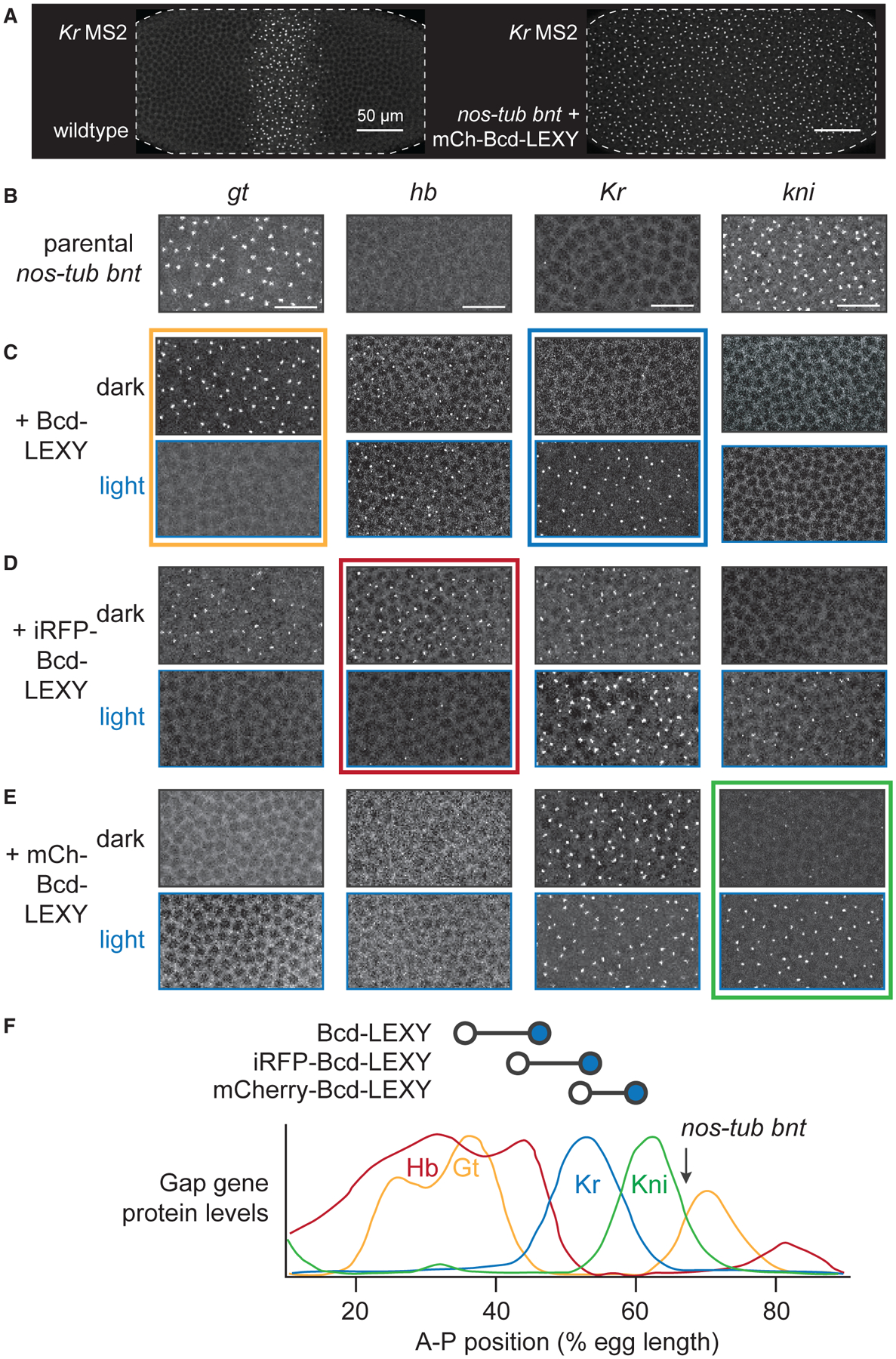
Spatially uniform, single-input embryos to enable optogenetic interrogation of specific gap genes (A) Nuclear cycle 14 embryos imaged using a *Kr* MS2 reporter. Left: embryo exhibiting wild-type A-P patterning. Right: *nos-tub* embryo harboring a single copy of uniformly expressed mCherry-Bcd-LEXY. The *nos-tub bnt* background eliminates all maternally supplied A-P patterns, so a uniformly expressed Bcd-LEXY construct produces a single approximate A-P position per embryo. (B–E) Regions of embryos showing MS2 reporter transcription for all four gap genes in (B) *nos-tub bnt*, (C) *nos-tub bnt* + Bcd-LEXY, (D) *nos-tub bnt* + iRFP-Bcd-LEXY, and (E) *nos-tub bnt* + mCherry-Bcd-LEXY embryos. For optogenetic illumination experiments, embryos were bathed in 450 nm light for 1 h. Scale bars represent 20 μm. (F) Mapping approximate embryonic positions represented by dark and light conditions in each genetic background. Bottom: diagram from Petkova et al. (2019) quantifying gap gene expression as a function of A-P position, with posterior = 100% EL. Top: diagram shows each optogenetic construct and its approximate position based on gap gene expression in light (open circle) and dark (blue circle). See also Figure S2 and Table S3.

Our goal is to use the fly embryo as a laboratory to measure stimulus-response functions for Bcd’s regulation of gap gene expression. We thus sought to simplify the experimental system, eliminating redundant inputs to the gap gene network as well as the complex spatial patterns found in wild-type embryos. Recent studies (Hannon et al., 2017; Ing-Simmons et al., 2021; Petkova et al., 2019) established genetic strategies for producing embryos that lack all known sources of anterior-to-posterior variation, and we used these triple-mutant *bcd*^*E1*^ *nos*^*BN*^ *tsl*^*4*^/*bcd*^*E1*^ *nos*^*l7*^ *tsl*^*4*^ (henceforth referred to as *bnt*) embryos as a starting point for introducing light-controlled Bcd-LEXY constructs (see STAR Methods for detailed information on fly stock and genetics). We also reintroduced uniform levels of a weak Nanos variant (*nos* TCEIIUC:AG) (Gavis et al., 2008; Gavis and Lehmann, 1994) to suppress maternal Hb protein expression (Figure S2A); the resulting embryos, termed *nos-tub bnt*, are devoid of all three A-P patterning cues and produce a posterior-like gene expression state throughout the embryo. Onto this background we expressed a single copy of the Bcd-LEXY construct at a uniform level across the embryo. To do so, we replaced the *bcd* 3′ UTR, which localizes this mRNA to the anterior pole and produces a protein gradient across the embryo, with the uniformly localized *sqh* 3′ UTR (Hannon et al., 2017). Bcd-LEXY *nos-tub bnt* embryos can thus be thought of as representing a single embryonic “position” set by the activity of the uniform Bcd-LEXY construct, which can subsequently be perturbed using light (see, e.g., Figure 3A for *Kr* MS2 in uniform mCherry-Bcd-LEXY embryos).

To define the A-P position represented by each uniformly expressed Bcd-LEXY construct, we measured the transcriptional activity for four gap genes (*gt*, *hb*, *Kr*, and *kni*) (see STAR Methods). We used previously published MS2 reporters for each gap gene in which multi-enhancer regulatory sequences (~20 kb upstream sequences for *hb* and *kni*, a 10 kb upstream sequence for *gt*, and the 4 kb CD1 + CD2 enhancer regions for *Kr*) drive expression of 24xMS2 stem loops followed by the *yellow* gene (Bothma et al., 2015; El-Sherif and Levine, 2016; Syed et al., 2021). We confirmed that each reporter closely matched the expected endogenous patterns of gap gene expression in embryos with intact A-P patterning (Figures S2B and S2C). We also verified that the *nos-tub bnt* background produced uniform levels and dynamics of these gap gene reporters at all embryonic positions, as expected from the removal of known A-P patterning cues (see, e.g., Figure S2D for *kni* MS2 in *nos-tub bnt* and *Kr* MS2 *nos-tub bnt* + mCherry-Bcd-LEXY).

We characterized the ground transcriptional state of gap gene MS2 reporters in our *nos-tub bnt* embryos and how it was perturbed by the addition of different optogenetic Bcd constructs. *nos-tub bnt* embryos transcribed high levels of *kni* MS2 and *gt* MS2, but low levels of *Kr* MS2 and no detectable *hb* MS2 (Figure 3B and arrow in 3F). This pattern was altered dramatically in the presence of uniformly expressed Bcd-LEXY, which drove an anterior-like transcriptional state of *gt* MS2 and *hb* MS2 in the dark, shifting to a mid-embryo-like state of *hb* MS2 and *Kr* MS2 transcription in the light (Figure 3C). Uniform iRFP-Bcd-LEXY embryos transcribed *gt* MS2, *hb* MS2, and *Kr* MS2 in the dark, shifting to *Kr* MS2 and *kni* MS2 expression in the light (Figure 3D). Finally, mCherry-Bcd-LEXY embryos shifted between weak and strong *kni* MS2 transcription depending on illumination conditions, with high *Kr* MS2 transcription in both cases (Figure 3E).

Comparing the combinations of gap genes expressed in each background to a wild-type embryo suggests a mapping between each Bcd-LEXY construct and embryonic position (Figure 3F). Illumination shifts Bcd-LEXY embryos from high transcription levels of *hb* MS2 and *gt* MS2 to *hb* MS2 and *Kr* MS2 expression, which can be mapped onto the expected gap gene pattern as a shift from ~35% to ~45% egg length (EL) upon illumination. Similarly, iRFP-Bcd-LEXY embryos shift from ~45% to 55% EL upon illumination, and mCherry-Bcd-LEXY embryos from ~50% to 60% EL (Figure 3F, top). Notably, parental *nos-tub bnt* embryos express high levels of *kni* MS2 and *gt* MS2, consistent with a position of ~70% EL, likely because the absence of Torso/Erk signaling at the termini prevents expression of more posterior targets (Figure 3F, arrow). Importantly, these results define different optogenetic Bcd constructs that can be used to switch each of the four core gap genes between high and low expression levels: *gt* (Bcd-LEXY), *hb* (iRFP-Bcd-LEXY), *Kr* (Bcd-LEXY), and *kni* (mCherry-Bcd-LEXY) (Figures 3B–3E; colored boxes), which we used to interrogate the transcriptional dynamics of each gap gene in the following live-imaging experiments.

### Anterior patterns of *hb* and *gt* respond rapidly to changes in nuclear Bcd concentrations

**Figure 4.**
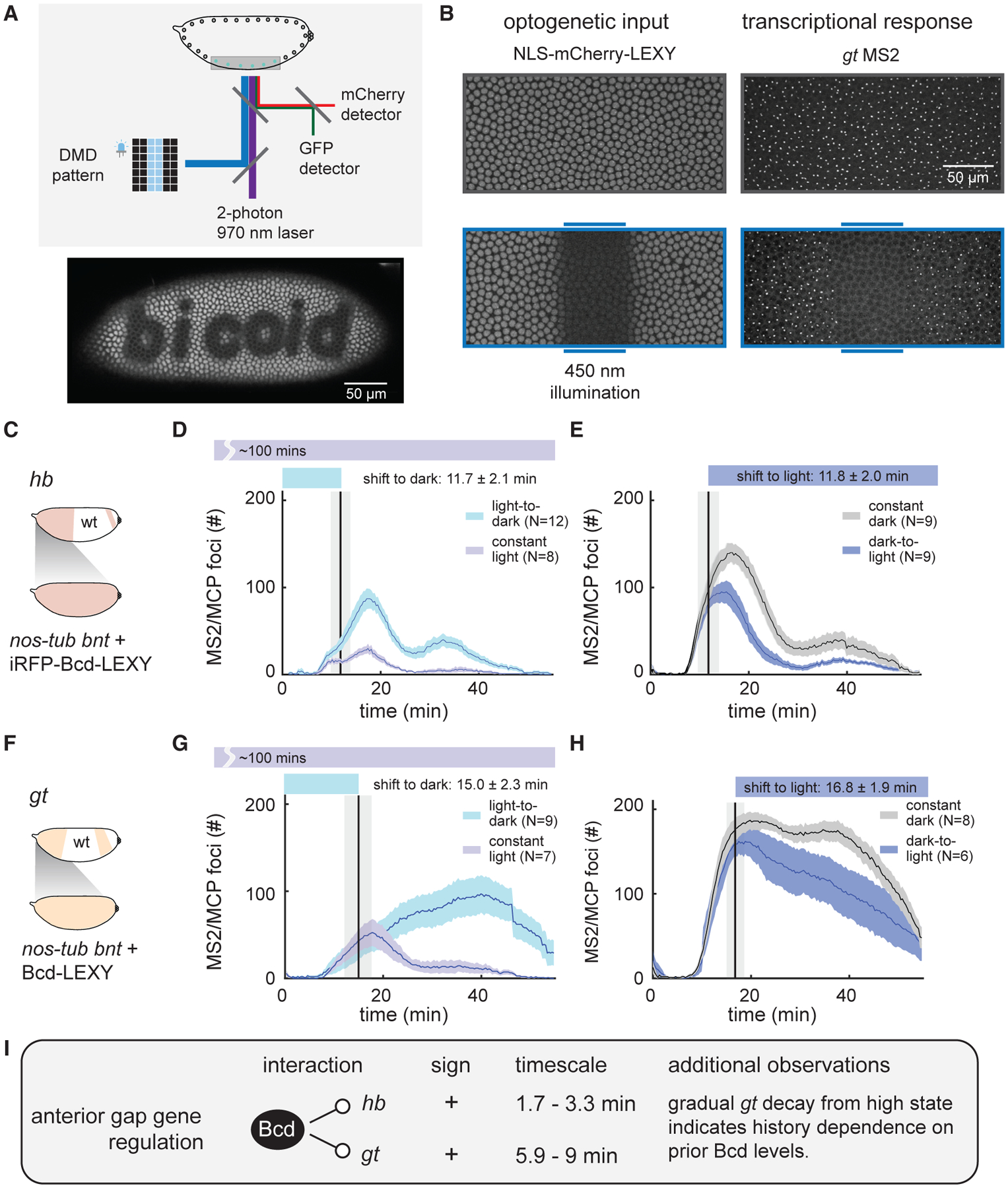
Optogenetic stimulation and live transcription measurement for anterior expression patterns of *gt* and *hb* (A) Schematic of optogenetic activation and two-photon imaging system. A 450 nm LED is patterned using a digital micromirror device (DMD) to deliver optogenetic stimuli. Two-photon imaging at 970 nm excites EGFP and mCherry without cross talk to the LEXY optogenetic system. (B) Example of light stimulation and two-color imaging of NLS-mCherry-LEXY and MCP/MS2 foci for a *gt* MS2 transcriptional reporter. Images show ventral regions of representative embryos in the absence or presence of a 450 nm light input delivered in a stripe in the middle of the embryo. (C–E) Optogenetic interrogation of Bcd-induced anterior *hb* transcription dynamics. Uniformly expressed iRFP-Bcd-LEXY embryos were imaged for *hb* MS2 reporter expression (schematic in [C]) upon an acute shift from light to dark (in [D]) and dark to light (in [E]); constant-light and constant-dark stimuli were used as controls. (F–H) Optogenetic interrogation of Bcd-induced anterior *gt* transcription dynamics. Uniformly expressed Bcd-LEXY embryos were imaged for *gt* MS2 reporter expression (schematic in [F]) upon an acute shift from light to dark (in [G]) and dark to light (in [H]); constant-light and constant-dark stimuli were used as controls. (I) Summary of stimulus-response results for *gt* and *hb*. Rapid light-triggered changes in both *gt* MS2 and *hb* MS2 transcription are consistent with direct activation by Bcd, and subsequent gradual changes in *gt* MS2 transcription suggest history-dependent transcription. For (D), (E), (G), and (H), shaded regions of transcriptional foci count show standard error of the mean, and the number of embryos tested is indicated on each plot. The vertical line indicates the mean time point when light stimuli change (either from dark to light or from light to dark) with a shaded region representing standard deviation. See also Figures S3–S6, Tables S2 and S3, and Video S2.

How do gap genes respond to acute changes in nuclear Bcd concentration? To answer this question, we set out to combine optogenetic Bcd-LEXY control with live imaging of individual gap genes using the MS2/MCP system. We constructed a confocal microscope that combines a tunable two-photon (2P) laser for GFP/mCherry imaging with a digital micromirror device and 450 nm LED for optogenetic stimulation (see STAR Methods; Figures 4A and S3A). The 2P excitation is ideal because it can be used for simultaneous GFP and mCherry imaging without triggering activation of AsLOV2-based optogenetic tools such as LEXY, due to the AsLOV2 domain’s blue-shifted 2P action spectrum relative to GFP (Homans et al., 2018; Kinjo et al., 2019). We confirmed that imaging EGFP at 970 nm resulted in negligible LEXY nuclear translocation (Figure S3B; Video S1), enabling high-resolution volumetric imaging without undesirable photoactivation of our optogenetic system. This technique should be broadly applicable to imaging EGFP in the presence of other AsLOV2-based optogenetic tools.

We engineered embryos that maternally express a desired uniform Bcd-LEXY construct as well as two additional constructs: an MCP-mNeonGreen protein for live transcript visualization (Bothma et al., 2015; Garcia et al., 2013; Lucas et al., 2013) and an NLS-mCherry-LEXY indicator to define the current activity state of our optogenetic system (Figures S3C and S3D; see STAR Methods). By crossing females of this genotype with males harboring a desired MS2-tagged gap gene reporter, we can thus deliver optogenetic stimuli while imaging both LEXY nuclear translocation and transcriptional responses in individual nuclei over time in live embryos (see Figures 4B and S4A using the *gt* MS2 reporter). When appropriate combinations of Bcd-LEXY constructs were co-expressed with gap gene MS2 reporters, we found that local illumination was sufficient to drive sharp boundaries of localized gene expression at any user-defined embryonic position (see Figures S4A–S4C for examples of *gt* MS2, *Kr* MS2, and *kni* MS2; Video S2). We observed that light-induced Bcd stimuli affected both the number and the intensity of transcriptional foci; we chose to quantify the number of transcriptional foci because it was more sensitive under conditions of low transcription (Figures S5A and S5B). We delivered all acute optogenetic perturbations during early NC14, a time when gap genes are normally highly transcribed.

We first performed stimulus-response measurements for Bcd regulation of *hb* transcription using our medium-activity iRFPBcd-LEXY construct (Figures 4C and 4D). We measured transcription of the *hb* MS2 reporter (Bothma et al., 2015) in response to an acute increase in Bcd activity by shifting from blue light to dark conditions; continuously illuminated embryos were used as a control (Figures 4D and S6A). We found that *hb* MS2 transcription rose rapidly after a light-to-dark shift; quantifying this response time revealed a shift within 1.7 ± 0.9 min after light perturbation (mean ± SEM; see Table S2, STAR Methods, and Figures S5C and S5D for response time calculation details). Conversely, acute removal of Bcd by switching from dark to light conditions caused *hb* MS2 transcription to fall relative to dark-incubated controls within 3.3 ± 1.1 min (Figure 4E). Similarly, rapid changes in transcription were also observed between illuminated and unilluminated regions within single embryos (Figures S5E and S5F). These data indicate that gap gene transcription can respond extremely rapidly to acute increases or decreases in nuclear Bcd concentration and are consistent with Bcd acting as a direct transcriptional activator of anterior *hb* expression.

How do the anterior expression dynamics of *gt* compare with those of *hb*? We examined the *gt* MS2 reporter (Syed et al., 2021) under similar light-to-dark and dark-to-light illumination shifts in NC14 (Figures 4F–4H and S6B). As in the case of *hb*, we found that optogenetic Bcd perturbations were rapidly transmitted to transcription of *gt* MS2, with response times of 5.9 ± 1.6 and 9 ± 2.7 min depending on the illumination sequence (Figures 4G and 4H; Table S2). However, unlike the case for *hb*, *gt* transcription changed only gradually, taking tens of minutes to approach transcription states similar to those of constant-stimulated controls. For example, dark-to-light shifted embryos (Figure 4H) continued to exhibit *gt* expression at levels considerably higher than constant-light embryos (Figure 4G) throughout the entirety of NC14. These data indicate a strong degree of history dependence on *gt* expression: current transcription depends on prior nuclear Bcd levels tens of minutes earlier. One mechanism by which such history dependence can arise could be if *gt* transcription were positively influenced by the past history of gap gene (*gt* or *hb*) expression (Alon, 2007), as suggested by a recent report of positive feedback on *gt* transcription by Gt protein (Hoermann et al., 2016). Nevertheless, our data do not rule out other mechanisms of history dependence. In summary, live-embryo stimulus-response measurements identify *hb* and *gt* as direct Bcd transcriptional targets and additionally suggest *gt* as a target of history-dependent regulation (Figure 4I).

### Optogenetic Bcd stimuli produce delayed and inverted *Kr* transcriptional responses

**Figure 5.**
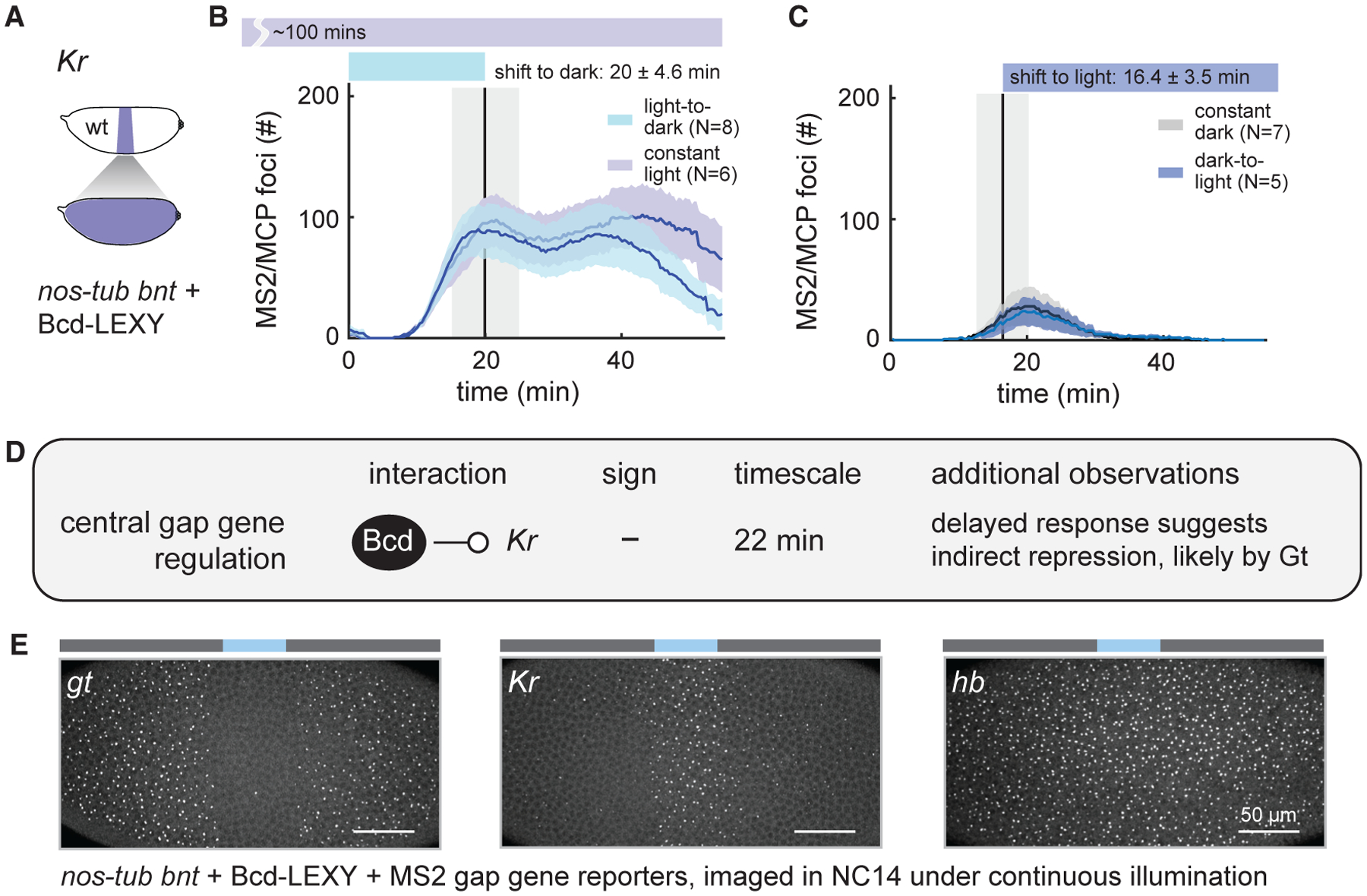
Acute perturbation of Bcd reveals delayed negative regulation of *Kr* expression (A–C) Optogenetic interrogation of Bcd-induced *Kr* expression dynamics. Uniformly expressed Bcd-LEXY embryos were imaged for *Kr* MS2 reporter expression (schematic in [A]) upon an acute shift from light to dark (in [B]) and dark to light (in [C]); constant-light and constant-dark stimuli were used as controls. (D) Summary of rapid perturbation results. An acute increase in Bcd-LEXY expression drives loss of *Kr* MS2 signal after a 22 min delay. Conversely, *Kr* MS2 transcription is not observed for at least 1 h after an acute decrease in nuclear Bcd levels. (E) Measurement of *gt*, *Kr*, and *hb* transcription after continuous, local illumination in Bcd-LEXY embryos. Transcription of *gt* is suppressed when *Kr* is transcribed, whereas *hb* is largely unaffected in the Bcd-LEXY background. For (B) and (C), shaded regions of transcriptional foci counts show standard error of the mean, and the number of embryos tested is indicated on each plot. The vertical line indicates the mean time point when light stimuli change (either from dark to light or from light to dark) with the shaded region representing standard deviation. See also Figures S4–S6, Tables S2 and S3, and Video S2.

To explore how dynamic changes in Bcd concentration alter the expression of gap genes in the middle of the embryo, we next turned to the gap gene *Kr* (Figure 5A). *Kr* expression is known to be regulated by multiple transcription factors, including Bcd (Hoch et al., 1991; Jacob et al., 1991; Struhl et al., 1992), Stat92E (Tsurumi et al., 2011), Zelda (Zld) (Nien et al., 2011), and Hb (Schulz and Tautz, 1994; Struhl et al., 1992); this complex regulation is thought to ensure that *Kr* is expressed in a narrow central band, with low expression at both anterior and posterior embryonic positions.

We performed stimulus-response measurements in Bcd-LEXY embryos, which exhibit stark differences in transcriptional activity of *Kr* MS2 between constant blue light and dark conditions that reflect optogenetic switching across the anterior boundary of the *Kr* pattern (Figure S6C). An acute increase in nuclear Bcd-LEXY drove a corresponding decrease in *Kr* MS2 transcription, consistent with our expectation of low *Kr* expression at anterior positions (Figure 5B). However, unlike *hb* and *gt*, the change in transcriptional activity of *Kr* MS2 began only after a 22 ± 2 min delay (Table S2). Conversely, shifting the embryo from a high-Bcd state to a low-Bcd state in early NC14 did not lead to any detectable change in *Kr* MS2 expression prior to gastrulation, indicating that an even longer time period may be required to establish *Kr* expression upon loss of Bcd (Figure 5C).

These results support a model whereby high nuclear Bcd levels induce expression of a stable repressor of *Kr* transcription (Figure 5D). An acute rise in Bcd would produce the repressor only after the time needed for new protein synthesis, and repressor degradation would be required for *Kr* transcription to respond to a drop in Bcd activity. Our data also point to a likely candidate repressor among the gap genes. We observe a tight correlation between the spatial expression domains of *Kr* MS2 and *gt* MS2 in illuminated Bcd-LEXY embryos, suggesting that Gt may act as the long-lived, Bcd-induced negative regulator (Figure 5E). This model is well supported by prior studies (Huang et al., 2020; Kraut and Levine, 1991b; Ochoa-Espinosa et al., 2005) identifying Gt as a potent repressor of *Kr* expression. In sum, our stimulus-response framework can be used to measure transcription dynamics that can in turn provide insight into direct and indirect links within a gene-regulatory network.

### *kni* is transcribed rapidly upon light-triggered loss of nuclear Bcd

**Figure 6.**
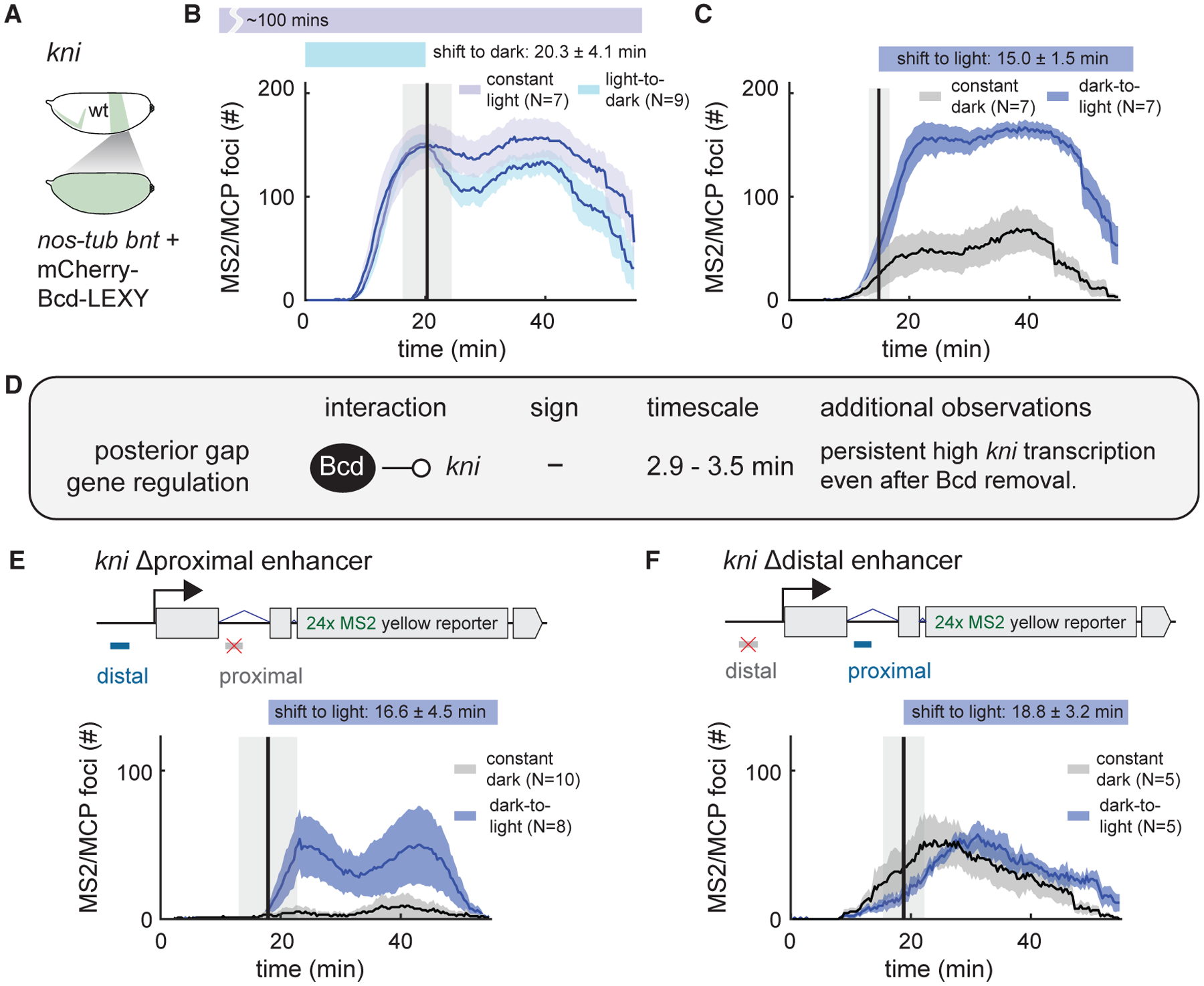
Acute removal of Bcd drives rapid activation of posterior *kni* expression (A–C) Uniformly expressed mCherry-Bcd-LEXY embryos were imaged using a *kni* MS2 reporter upon an acute shift from light to dark (in [B]) and dark to light (in [C]); constant-light and constant-dark stimuli were used as controls. (D) Summary of rapid perturbation results. An acute decrease in mCherry-Bcd-LEXY expression, representing a change from central to posterior Bcd levels, drives a rapid rise in transcriptional activity of *kni* MS2 reporter. In the converse experiment, *kni* MS2 transcription drops rapidly but only slightly upon acute Bcd nuclear import, suggesting that *kni* transcription may be positively autoregulated. (E and F) Experiments as in (C) for *kni* MS2 reporters in which the proximal enhancer (in [E]) or the distal enhancer (in [F]) was replaced with nontargeted sequence. For (B), (C), (E), and (F), shaded regions of transcriptional foci counts show standard error of the mean, and the number of embryos tested is indicated on each plot. The vertical line indicates the mean time point when light stimuli change (either from dark to light or from light to dark), with the shaded region representing standard deviation. See also Figures S4–S6, Tables S2 and S3, and Video S2.

Our final target for optogenetic stimulus-response analysis was the posterior pattern of *kni* transcription (Figure 6A). The gap gene *kni*, which is required for specification of posterior body segments, is thought to be induced by Bcd and Caudal (Cad) and repressed by Hb (Hulskamp et al., 1990; Rivera-Pomar et al., 1995). This complex and redundant regulation involves both maternally supplied anterior inputs (e.g., Bcd-dependent Cad patterning) and posterior cues (e.g., Nanos-dependent patterning of maternal Hb). Interestingly, we observe high *kni* MS2 signal even in *nos-tub bnt* embryos (Figure 3B), raising the question of how Bcd affects expression of a gap gene that is still highly expressed in the absence of Bcd.

We examined the Bcd-dependent dynamics of *kni* MS2 transcription (Bothma et al., 2015) in embryos expressing the lowest-activity mCherry-Bcd-LEXY construct (Figures 6B, 6C, and S6D). Acutely dropping nuclear Bcd concentration led to a dramatic and unexpected change in *kni* MS2 transcription (Figure 6C). Within 2.9 ± 0.9 min after a loss of nuclear Bcd, *kni* MS2 transcription began rising rapidly to levels that were comparable to those achieved under continuous illumination (Figure 6B). Conversely, a light-induced increase in nuclear Bcd triggered a similarly rapid but smaller-amplitude decrease in *kni* MS2 transcription (Figure 6B). Just as in the case of *gt*, the stability of the high-*kni*-expressing state may be indicative of positive autoregulation of *kni* expression by its own protein product. Together, these data suggest that Bcd can act as an apparent repressor of *kni* expression, an unexpected role for Bcd, which is typically considered to perform only transcriptional activation functions. The initiation of *kni* transcription within 2 min after Bcd nuclear export is compatible only with a direct regulatory link, not Bcd-induced expression of an intermediate repressor.

To gain further insight into the repressive effect, we set out to define its requirements in the *kni* enhancer regions. The posterior pattern of *kni* expression is known to be regulated by two enhancers, an 818 bp proximal enhancer and a 2.3 kb distal enhancer (Li et al., 2021; Rivera-Pomar et al., 1995). We generated embryos expressing *kni* MS2 reporters with either the proximal or the distal enhancer sequence replaced with nonregulated sequence (Bothma et al., 2015) and monitored the MS2 signal in response to acute Bcd removal (Figures 6E and 6F). We found that the *kni* reporter lacking the proximal enhancer (*kni* Δproximal reporter) still showed potent regulation by mCherry-Bcd-LEXY, whereas the *kni* Δdistal reporter was not affected by light-induced changes in nuclear Bcd (Figures S6E and S6F; Video S3). Transcription from the *kni* Δproximal reporter also rose rapidly upon the shift to blue light, matching what was observed from the wild-type regulatory sequence (Figures 6C and 6E). Our results are consistent with prior observations that the *kni* distal enhancer exhibits higher Bcd binding than does the *kni* proximal enhancer, arguing that Bcd exerts its regulatory effects at the distal enhancer (Rivera-Pomar et al., 1995). In summary, our acute stimulus-response framework identifies a rapid, repressive role for Bcd in regulating *kni* transcription through the *kni* distal enhancer, highlighting the power of optogenetic perturbation in a simplified genetic context to identify both known and unknown gene-regulatory relationships.

## DISCUSSION

### A stimulus-response strategy for dissecting complex developmental gene networks

We have described a combined genetic and optogenetic strategy to gain insight into a canonical developmental patterning system: the control of gap gene expression by the Bcd morphogen during *Drosophila* embryogenesis. Our strategy relies on three advances. First, we experimentally simplify the conditions under which the gap gene network operates, eliminating all pre-existing landmarks along the A-P axis to produce embryos with uniform positional identity. Although the reduced network involves just one input transcription factor (Bcd) and four output genes (anterior *giant*, anterior *hunchback*, central *Krüppel*, and posterior *knirps*), it captures much of the complexity of the wild-type pattern, including stripes of gap gene expression when Bcd is delivered in a head-to-tail gradient (Briscoe and Small, 2015; Petkova et al., 2019). Second, we reintroduce optogenetic Bcd constructs to shift these uniform embryos to any of three distinct A-P positions, enabling us to experimentally isolate specific gap gene patterns. Finally, we combine acute optogenetic perturbation with live-cell biosensors of target gene expression to map each target gene’s response to acute changes in transcription factor concentration over time. Doing so required establishing new imaging methods for two-color confocal imaging and optogenetic activation *in vivo*, a challenge we solved by combining 970 nm 2P imaging of GFP/mCherry with 450 nm excitation of the LEXY optogenetic system.

### Bcd-dependent regulation of anterior and posterior gap gene patterns

Our optogenetic stimulus-response experiments broadly support the canonical view of Bcd as a transcriptional activator of *gt*, *hb*, and *Kr*. We find that both *gt* and *hb* are transcribed rapidly upon acute Bcd nuclear import (Figure 4), and *Kr* transcription is absent in *bnt nos-tub* embryos but present when low Bcd activity is introduced on top of this background (Figure 3). Our data also point to multiple regulatory links between gap genes. We find that both *gt* and *kni* exhibit strong history dependence, responding rapidly but incompletely after a shift to light conditions that should elicit low transcription of these gap genes (Figures 4 and 6) (Astrid et al., 2016; Jaeger et al., 2004a) We also find that *Kr* exhibits delayed negative regulation by Bcd (Figure 5), likely through Gt as an intermediate node (Huang et al., 2020; Kraut and Levine, 1991a; Ochoa-Espinosa et al., 2009). Importantly, each of these network connections can be identified using a single, unified experimental workflow: acute optogenetic Bcd perturbation and live recording and quantification of a target gene’s transcriptional dynamics.

Our study also revealed an unexpected result: a rapid increase in *kni* transcription after acute removal of mCherry-Bcd-LEXY from the nucleus. Bcd is not expected to act as a transcriptional repressor, so it is surprising to find any context in which its removal triggers rapid initiation of transcription. Classical models interpret the absence of posterior *kni* at anterior positions as being the consequence of indirect Bcd-dependent regulation: repression by anterior gap gene products (e.g., *hb* or *Kr*) or weak activation by Cad, which is translationally repressed by Bcd (Niessing et al., 2002; Pankratz et al., 1992; Rivera-Pomar et al., 1995). Our result appears inconsistent with all of these explanations, as *kni* MS2 transcription rises near instantaneously after mCherry-Bcd-LEXY nuclear export (Figure 6), too rapidly for changes in gap gene or Cad protein levels to occur. Furthermore, rapid *kni* derepression requires the distal enhancer, the predominant site of Bcd binding (Li et al., 2021; Rivera-Pomar et al., 1995) It is still incompletely understood how different Bcd concentrations specify both anterior and posterior positional identity (Hannon et al., 2017); a clearer understanding of how Bcd-dependent repression of *kni* is achieved may clarify how low Bcd concentrations can be accurately sensed even at posterior positions.

How might rapid transcriptional activation occur upon loss of nuclear Bcd? Our data are consistent with many possible mechanisms. Bcd may compete for binding to the *kni* distal enhancer with another more potent transcriptional activator, such that Bcd loss paradoxically increases *kni* transcription. Alternatively, Bcd may cooperatively associate with a transcriptional repressor at the *kni* enhancer and lead to increased repressor binding, mirroring the well-established interaction between Dorsal and Groucho for repressing subsets of genes along the dorsoventral axis (Lehming et al., 1994; Dubnicoff et al., 1997). We look forward to future studies that precisely define Bcd’s repressive role in *kni* transcription, as well as the extension of our acute stimulus-response methods to other transcription factor (TF)-target gene pairs in complex regulatory networks.

Optogenetic stimuli have recently found widespread use in developmental contexts, from identifying critical time windows for developmental decisions (Huang and Saunders, 2020; Di Pietro et al., 2021; Johnson et al., 2017; Huang et al., 2017; Kogler et al., 2021; Sako et al., 2016; McDaniel and Harrison, 2019; Viswanathan et al., 2021) to erasing and replacing signaling gradients with spatial light patterns (Johnson et al., 2020). Here we show that optogenetics can be used at a more granular level to home in on dynamic relationships between a TF and its target genes *in vivo*. Nevertheless, work on an experimentally reduced system constitutes only a first step in understanding the full gap gene network, and we look forward to future studies that examine Bcd-dependent responses as other factors from the natural system are systematically reintroduced. We can also envision extending the current approach to perturbing multiple nodes (e.g., by constructing LEXY fusions of all gap genes), and coupling these approaches to quantitative modeling (Jaeger et al., 2004b) could elaborate network architecture still further. The future is bright for optogenetic interrogation of developmental gene networks.

### Limitations of this study

Our study reports a series of Bcd-LEXY constructs that are useful for probing gap gene transcription at different embryonic positions, but the mechanistic basis for these activity differences is still unknown. While we do observe some expression-level differences between variants, they are unlikely to explain the large change in activity between the Bcd-LEXY and the mCherry-Bcd-LEXY constructs. Future work should explore alternative methods to vary optogenetic Bcd activity (e.g., gene dosage, protein stability, or promoter strength). A second limitation is that we primarily consider Bcd’s role as a TF, not its additional role as a translational repressor of Cad protein. Alternative optogenetic strategies and live-cell Cad biosensors (Rödel et al., 2013) could also be used to dissect the dynamics of Cad regulation. Finally, we note that the LEXY system could be further improved, perhaps by combining strategies to increase its dynamic range with those that maintain rapid switching kinetics. Such a system might yet achieve the grand goal of recapitulating the full range of Bcd doses in a single embryo using optically patterned gradients.

## STAR★METHODS

### RESOURCE AVAILABILITY

#### Lead contact

Further information and requests for resources and reagents should be directed to and will be fulfilled by the lead contact, Jared Toettcher (toettcher@princeton.edu).

#### Materials availability

All materials generated in this study will be provided upon request. Plasmids encoding the Bcd-LEXY, IRFP-Bcd-LEXY, mCherry-Bcd-LEXY and NLS-mCherry-LEXY inserts are available from the Addgene repository.

#### Data and code availability

All data and analyses reported in this paper will be provided by the lead contact upon request. MATLAB scripts for the analysis of MCP/MS2 transcription foci, nuclear intensity, and spatial pattern have been deposited at Zenodo and are publicly available as of the date of publication. DOIs are listed in the Key Resources Table.

Any additional information required to reanalyze the data reported in this paper is available from the Lead Contact upon request.

### EXPERIMENTAL MODEL AND SUBJECT DETAILS

*Drosophila melanogaster* lines (see Key Resources Table) were raised at 25°C. For live imaging of embryos, collection cages were kept in dark, and corresponding stimulation conditions (see Method Details) were applied while imaging on the microscope. For fixation and immunostaining, light conditions were described in Method Details.

### METHOD DETAILS

#### Plasmids

Constructs were generated using In-Fusion assembly (Clontech) and oligonucleotides for primers were obtained from Integrated DNA Technologies. Constructs are available via Addgene or on request. Bcd-LEXY constructs are generated from pCol-a*Tub67C-*EGFP-Bcd-FRT-*bcd* 3’UTR- 3xP3-RFP-FRT-*sqh* 3’UTR (Hannon et al., 2017) where the N-terminal EGFP was either removed or replaced by iRFP or mCherry and LEXY domain was inserted as C-terminus with a 15 aminoacid long linker in between. NLS-mCherry-LEXY constructs are generated by ligation of the NLS-mCherry-LEXY insert part PCR amplified from a mammalian expression vector Addgene #72655 (Niopek et al., 2016) and a fly expression vector pBabr-mTub-MCS-*sqh*3’UTR (courtesy from Wieschaus lab) digested by restriction enzymes NheI and SpeI. mCherry was subsequently replaced by EGFP to generate NLSEGFP-LEXY plasmid.

#### Fly stocks and genetics

##### Establishing Bcd-LEXY and bcd nos tsl fly stocks

For generation of transgenic flies and stocks, all four Bcd-LEXY (Bcd-LEXY, EGFP-Bcd-LEXY, iRFP-Bcd-LEXY and mCherry-Bcd-LEXY) constructs were integrated into the third chromosome using the φC31-based integration system (Bischof et al., 2007) at the VK33 site (65B2) by BestGene. NLS-mCherry-LEXY and NLS-EGFP-LEXY constructs were integrated into the second chromosome at the VK02 site (47C6). Each Bcd-LEXY construct was then further recombined either with *bcd*^*E1*^ or *bcd*^*E1*^ *nos*^*BN*^ *tsl*^*4*^ on the third chromosome (Hannon et al., 2017; Petkova et al., 2019) NLS-mCherry-LEXY was recombined with MCP-mNeonGreen on the second chromosome, and further crossed with *bcd*^*E1*^ *nos*^*l7*^ *tsl*^*4*^ on third chromosome to generate MCP-mNeonGreen NLS-mCherry-LEXY/Cyo; *bnt*/TM3 flies.

We obtained few and poor-quality embryos from *bcd*^*E1*^ *nos*^*BN*^ *tsl*^*4*^ homozygous females, and thus used *bcd*^*E1*^ *nos*^*BN*^ *tsl*^*4*^*/bcd*^*E1*^ *nos*^*l7*^ *tsl*^*4*^ transheterozygotes for further experiments. While *nos*^*BN*^ is a complete loss of both *nos* RNA and protein, which impedes both pole cell migration and therefore germline cells formation and abdominal segmentation, *nos*^*l7*^ is a partial deletion near C-terminal of the zinc-finger domain that maintains normal germline development. However, in terms of body segmentation phenotype and gap gene expression pattern, *nos*^*BN*^ and *nos*^*l7*^ both exhibit indistinguishable patterns as expected from severe loss of function (Lehmann and Arrizabalaga Muñiz, 1999; Asaoka et al., 1998) supporting the use of this transheterozygous background for *nanos* loss of function in A-P patterning.

##### Establishing uniform Bcd-LEXY embryos

To achieve uniform Bcd expression, Bcd-LEXY, *bnt* flies were crossed to heat shock-inducible *flippase* expressing flies and the resulting larvae were heat shocked at 37°C for three continuous days for 1 h each day. After one generation of outcrossing, progeny lacking the *bcd* 3’UTR were sorted by loss of RFP expression. Then Bcd-LEXY constructs were driven by *sqh* 3’UTR resulting in a uniform distribution of Bcd along the AP axis (Hannon et al., 2017).

##### Establishing nos-tub bnt uniform Bcd-LEXY embryos

In wild-type embryos, *nanos* mRNA is localized at the posterior pole and produces a posterior-to-anterior gradient of Nanos protein (Gavis and Lehmann, 1994) A second population of *nanos* mRNA is not asymmetrically patterned and produces uniform Nanos protein that plays a crucial role in suppressing maternal Hunchback translation (Gavis et al., 2008) Complete loss of *nanos* disrupts both the patterned and uniform contributions, leading to abnormally high levels of maternal Hunchback throughout the embryo. We thus used a *nos-tub*:TCEIIUC:AG construct (Gavis et al., 2008) (courtesy of the Gavis lab) as a uniformly-expressed, reduced-activity form of Nanos to reduce maternal Hb levels, thereby allowing expression of abdominal gap genes like *kni* and *gt*. The *nos-tub* construct was further recombined to the *Sp* marker on the same chromosome (2^nd^ chromosome) to mark the transgene (hereafter referred as *Sp*, *nos-tub*), and then crossed with male uniform Bcd-LEXY *bcd*^*E1*^*, ri, nos*^*BN*,^ *e, tsl*^*4*^/TM3, *sb, ri* flies to generate *Sp*, *nos-tub*/+; uBcd-LEXY *bcd*^*E1*^*, ri, nos*^*BN*^*, e, tsl*^*4*^ / TM3, *sb, ri* flies. By crossing males of the preceding genotype to MCP-mNeonGreen, NLS-mCherry-LEXY / Cyo; *bcd*^*E1*^*, ri, nos*^*l7*^ *tsl*^*4*^ / TM3*,sb, ri* females, we selected female flies with *Sp* and *ri* markers to ensure the correct genotype of *Sp*, *nos-tub* / MCP-mNeonGreen NLS-mCherry-LEXY; uBcd-LEXY *bcd*^*E1*^*, ri, nos*^*BN*^*, e, tsl*^*4*^ / *bcd*^*E1*^*, ri, nos*^*l7*^ *tsl*^*4*^ that we then caged with homozygous MS2 reporter males for live gap gene transcription imaging.

##### MS2 reporters used in this study

*hb* BAC>MS2 (BAC CH322–55J23) and *kni* BAC>MS2 (BAC CH322–21P08) were described in Bothma *et al*., where CHORI BACs (~21kb) were used as starting points, with *kni* and *hb* coding sequences replaced with a 24xMS2-yellow-kanamycin reporter gene, leaving the 5’UTR and 3’UTR intact. *kni* Δdistal and Δproximal MS2 reporters were also described previously (Bothma et al., 2015), and were based on *kni* BAC>MS2 with the distal or proximal enhancer replaced by a fragment of lambda phage DNA. *Kr (CD1+CD2)*>MS2 was described in El-Sherif et al., where a 4kb upstream regulatory region including promoter region is fused to 24xMS2-yellow reporter (El-Sherif and Levine, 2016). *gt*>MS2 was generated using a 10kb upstream region of *giant*, including its promoter region, to drive 24xMS2-yellow reporter (Syed et al., 2021).

#### Cuticle preparation

For dark and light conditions, embryos with specific Bcd-LEXY constructs were collected between 0–1 h post laying in the dark on an agar plate. Then embryos for the light condition were placed under a custom-built panel of blue LEDs and removed from light after 4hrs. In dark conditions, embryos were kept in a light-tight box away from ambient room light or blue light to prevent inadvertent optogenetic stimulation. After a 3 h incubation in light or dark conditions, embryos were kept at room temperature (at 22°C) for another 24–36 h and then bleached, then moved to the methanol-heptane glass tube and vigorously shaken for 20 s. Embryos settled at the bottom were removed and placed on a glass slide with Hoyer’s solution (1:1 premix lactic acid) and sandwiched between the glass slide and cover glass. The slide was placed at 65°C overnight and then imaged on a Nikon Eclipse Ni dark-field microscope at 10x zoom.

#### Immunostaining and imaging

Embryos were collected every 2 h and aged in dark for another 2 h. Embryos were dechorionated by bleaching and heat fixed in dark, and then stained essentially as described in (Hannon et al., 2017; Petkova et al., 2019) with rabbit anti-Bcd, mouse anti-Hb primary antibodies (courtesy by Eric Wieschaus) and sheep anti-GFP (Invitrogen, USA) followed by fluorophore-conjugated secondary antibodies Alexa 488 (sheep), Alexa 594 (mouse), and Alexa 647 (rabbit) from Invitrogen. For pairwise comparisons of wild-type and mutant backgrounds, embryos expressing HisGFP collected the same way were mixed in each tube for staining and imaging. Stained embryos were imaged on a Nikon A1R laser-scanning confocal microscope, and a 5 μm z-stack around the midsagittal plane with step size of 1 μm were taken.

#### Two-photon microscopy

A custom microscope was built to simultaneously perform two-photon excitation imaging and localized optogenetic stimulation on the same setup. A Chameleon Ultra II tunable laser was used at 970 nm to simultaneously excite green and red-tagged biomolecules (Gregor et al., 2007; Svoboda et al., 1997) The laser beam was collimated and passed through a laser power modulator Pockels cell (350–80-LA-02 KD P Series E-O Modulator, Conoptics, USA). The output laser beam was expanded to 4 mm diameter (AC254–050-AB-ML, AC254–150-AB-ML, Thorlabs, USA) before reaching a two-axis scan galvo mirror (6210H, Cambridge Technology, USA). After the scan mirrors, the laser beam passed through an f-theta lens (focal length 63 mm; 4401-388-000-20, Linos, USA), a tube lens (focal length 180 mm, AC508–180-AB-ML, Thorlabs) and focused on the imaging sample using a high numerical aperture objective (NA, Nikon 1.3 NA, 40X). The fluorescence signal was collected and sent to two sensitive point photo multiplier tubes (H10770A-40, Hamamatsu, Japan). The microscope setup interfaces via data acquisition cards (DAQ; PCIe 6321 and PCIe 6374, National Instruments, USA) using MATLAB-based ScanImage 5.6 software (Pologruto et al., 2003) For live imaging, embryos were imaged close to cover glass surface (image resolution 1024 × 512 pixels at 3.2 μs pixel dwell-time; see imaging details in Table S3).

#### Optogenetic stimulation

LEXY perturbation was achieved using a digital micro-mirror device (DMD; DLP 4500 LightCrafter, Texas Instruments, USA) to project spatial patterns and to rapidly change light levels (Rullan et al., 2018; Wilson et al., 2017) (see Figure S3A) through a parallel light path using a long-pass 473 nm dichroic mirror and a combination of color and interference filters to attenuate the DMD’s blue LED wavelength (445 ± 8 nm). To synchronize two-photon image acquisition and DMD blue light activation cycles, an external trigger mode in DLP LightCrafter control software was used. The software controls the LED light wavelength, pulse duration, pulse duty cycle, the number of pulses, and the type of spatial image pattern to project on the imaging sample (see details in Table S3). The optimum blue light level for optogenetic perturbation was determined by optimizing the maximum protein export with minimal light scattering to neighboring nuclei (see Figures 1B and 1C). After scanning the range between 50 and 250 μW/cm^2^ of blue-light on/off pulsatile cycles of LEXY-tagged protein nuclear signal (data not shown), 100 μW/cm^2^ (pulse duration = 40 ms, pulse duty cycle = 100 ms, number of pulses = 5) was determined for all optogenetic perturbations performed in this study.

#### Live imaging data collection

For the live data acquisition and light perturbation experiments, flies were kept in dark at 25°C and the embryos were collected on an agar plate between 1 and 2 h post laying. For live imaging, embryos were dechorionated on double-sided tape and mounted on a glued membrane film (Lumox film, Starstedt, Germany), covered in halocarbon oil 27 and sandwiched between the membrane and the cover glass slide (cover glass washed and cleaned with pure ethanol, slide #1.5, Sigma BR470045). The data collection was performed using a custom-built two-photon microscope using 970 nm laser excitation for green (EGFP and mNeonGreen tagged proteins) and red (NLS-mCherry-LEXY) at room temperature (ranging from 21.5–22.5°C). The blue light perturbation was performed using a digital micromirror device (DMD) unit installed on the same system (see Two-photon Microscopy section above). Details on data collection for specific experiments are summarized in Table S3.

### QUANTIFICATION AND STATISTICAL ANALYSIS

#### Bcd-LEXY activity

To estimate the functional Bicoid activity (potency) of fluorescently tagged Bcd-LEXY fusion proteins (as well as remaining activity in *bcd*^*E1*^ homozygous mutant fly lines and the Bcd dose level), we used protein immunostaining of the Bcd target gene Hb and measured position shifts in the posterior Hb boundary as well as position shifts of the cephalic furrow. All shifts were scaled according to embryo length and the quantified estimates are presented in Figures 2D and 2E (Table S1 for cephalic furrow position shifts). Custom MATLAB imaging analysis code recognized the contour of the embryo and extracted the intensity of the surface nuclei for all three channels. Intensities of three channels were normalized to HisGFP embryos that mixed in each slide respectively, with 1 being the mean maximal intensity of HisGFP embryos. For Hb level, min-max normalization was further conducted for clear comparison of boundary position, and half maximal positions of the posterior boundary of anterior expression domain were picked out for each genotype for the boxplot.

#### LEXY tagged protein export and import kinetics

Blue light-induced LEXY export kinetics and nuclear localization signal (NLS)-induced import kinetics were determined by analyzing the nuclear intensity of the fluorescent moiety of these fusion proteins. Intensity time traces were averaged and fitted with a single exponential $n0*{\text{exp}}^{\left(-\frac{t}{\tau }\right)}$ to estimate the export rate, i.e. the inverse of the time constant. Similarly, the import time constants were estimated using $n0*\left(1-{\text{exp}}^{\left(-\frac{t}{\tau }\right)}\right)$ as the fitting model (Figures 2F–2H). Note: the uniform EGFP-Bcd-LEXY, NLS-mCherry-LEXY, and NLS-EGFP-LEXY lines were measured using the DMD-equipped custom-built two-photon microscope, while the uniform mCherry-Bcd-LEXY and iRFP-Bcd-LEXY lines were imaged on a commercial Nikon A1R confocal microscope (imaging conditions can be found in Table S3). For LEXY translocation kinetics fits, the mean and the standard deviation are presented for multiple embryo replicates.

#### Quantification of the reporter gene MS2 spots

We created a custom MATLAB script to analyze and visualize time-lapse MS2 counts. Two-color raw images were first acquired as TIFF files (see Table S3, Figure S4–S6). Nuclei were segmented from the NLS-mCherry-LEXY image channel. These intensity traces were used to estimate the timing of each nuclear cycle because nuclear envelope breakdown resulted in a profound loss of NLS-mCherry-LEXY intensity. The subsequent rise in NLS-mCherry-LEXY intensity upon nuclear envelope re-formation was set to 5.6 min in NC14 (see Figure S3D). For MS2 data, the image data was z-max projected (8 total z slices, each 1.1 μm apart), then a 2D Gaussian filter was applied to filter out small structures and followed by threshold to select MS2 spots. We quantified transcriptional activity from the total number of MS2 foci in a 40 × 150 μm^2^ ROI after thresholding in the center of the embryo; similar results were also obtained if the mean intensity of foci was calculated instead, albeit with greater noise when the number of detected foci was low (Figures S5A and S5B). All the data representing spot count time traces indicate the mean and standard error of the mean over multiple embryo measurements, unless stated otherwise.

#### Mean response time post light perturbation

The response time workflow is shown in Figures S5C and S5D, taking one *hb* MS2 light-to-dark shift as a representative example. For response time quantification, we measured the difference between the number of MS2 foci in each embryo’s illuminated region (e.g., after the light-to-dark shift) from the mean of all embryos in the corresponding control condition (e.g., under constant light) (Figure S5C). The resulting difference curves for each embryo were then smoothened (Garcia, 2010) and differentiated (Figure S5D). The “response time” was taken to be the time of maximum derivative, corresponding to the time point at which light stimulation diverged maximally from the unstimulated control. The response times for each embryo were used to obtain the mean +SEM reported in Table S2. We found that some embryos were non-responsive, possibly due to a late start or undesired illumination during setup, making response time calculations impossible. We thus excluded embryos whose maximum change in MS2 foci was less than 25% of the corresponding peak in control embryos. The number of excluded embryos is indicated for each condition in Table S2.

## Supplementary Material

1

Video S1

Video S2

Video S3

## Figures and Tables

**Table:** KEY RESOURCES TABLE

REAGENT or RESOURCE	SOURCE	IDENTIFIER
Antibodies		
Bcd rabbit antibody	Wieschaus lab	N/A
Hb mouse antibody	Wieschaus lab	N/A
GFP sheep antibody	Bio-Rad	Cat # 4745–1051; RRID:AB_619712
Donkey anti sheep, Alexa-488	Invitrogen	Cat # A-11015; RRID:AB_141362
Donkey anti mouse, Alexa-594	Invitrogen	Cat # R37115; RRID:AB_2556543
Donkey anti rabbit, Alexa-647	Invitrogen	Cat # A32795; RRID:AB_2762835
Bacterial and virus strains		
Stellar Chemically Competent Cells	ClonTech Laboratories	Cat # 636763
Chemicals, peptides, and recombinant proteins		
ClonAmp HiFi PCR polymerase	ClonTech Laboratories	Cat # 639298
PrimeSTAR GXL DNA Polymerase	ClonTech Laboratories	Cat # R050B
inFusion HD cloning kit	ClonTech Laboratories	Cat #638911
Halocarbon Oil 700	Sigma	Cat # H8898
Halocarbon Oil 27	Sigma	Cat # H8773
37% Formaldehyde solution	Sigma	Cat # F8775
Heptane	Sigma	Cat # 34873
Methanol	Sigma	Cat # 34860
PBS	Gibco	Cat # 14190144
Hoyer’s solution	Wieschaus lab	N/A
Qiagen miniprep kits	Qiagen	Cat #27106
NucleoSpin gel and PCR clean-up kits	ClonTech Laboratories	Cat # 740609
Experimental models: Organisms/strains		
*bod*^*E1*^ *nos*^*BN*^ *tsi*^*4*^	Wieschaus lab	N/A
*bod*^*E1*^ *nos*^*L7*^ *tsi*^*4*^	Wieschaus lab	N/A
Sp, nos-tub	Wieschaus lab, Gavis lab	N/A
nos>φ NLS-MCP-mNeonGreen	This study	N/A
αTub67C> NLS-mCherry-LEXY	This study	N/A
αTub67C> NLS-eGFP-LEXY	This study	N/A
αTub67C> eGFP-Bcd-LEXY FRT bcd 3’ UTR hsp70 RFP FRT sqh 3’UTR	This study	N/A
αTub67C> Bcd-LEXY FRT bcd 3’ UTR hsp70 RFP FRT sqh 3’UTR	This study	N/A
αTub67C> iRFP-Bcd-LEXY FRT bcd 3’ UTR hsp70 RFP FRT sqh 3’UTR	This study	N/A
αTub67C> mCherry-Bcd-LEXY FRT bcd 3’ UTR hsp70 RFP FRT sqh 3’UTR	This study	N/A
HisGFP	Bloomington	Cat # 5941
CyO; TM3, sb	Bloomington	Cat # 59967
*hb* BAC > MS2	(Bothma et al., 2015)	N/A
*kni* BAC > MS2	(Bothma et al., 2015)	N/A
*kni* ΔDistal > MS2	(Bothma et al., 2015)	N/A
*kni* ΔProx> MS2	(Bothma et al., 2015)	N/A
*Kr* (CD1+CD2) > MS2	(El-Sherif and Levine, 2016)	N/A
*gt* > MS2	(Syed etal., 2021)	N/A
Recombinant DNA		
αTub67C> eGFP-Bcd FRT bcd 3’ UTR hsp70 RFP FRT sqh 3’UTR	(Hannon et al., 2017)	N/A
αTub67C> Bcd-LEXY FRT bcd 3’ UTR hsp70 RFP FRT sqh 3’UTR	This paper	Addgene #182594
αTub67C> iRFP-Bcd-LEXY FRT bcd 3’ UTR hsp70 RFP FRT sqh 3’UTR	This paper	Addgene #182595
αTub67C> mCherry-Bcd-LEXY FRT bcd 3’ UTR hsp70 RFP FRT sqh 3’UTR	This paper	Addgene #182596
αTub67C> eGFP-Bcd-LEXY FRT bcd 3’ UTR hsp70 RFP FRT sqh 3’UTR	This paper	N/A
αTub67C> NLS-mCherry-LEXY	This paper	Addgene #182597
αTub67C> NLS-eGFP-LEXY	This paper	N/A
Software and algorithms		
MATLAB R2021a	MathWorks	RRID: SCR_001622
Python 3.10	Python Programming Language	RRID:SCR 008394
Fiji	(Schindelin et al., 2012)	http://fiji.sc; RRID: SCR_00228
National Instruments/Labview	National Instruments Corp.	RRID:SCR_014325
DLP 4500 LightCrafter control software	Texas Instruments	N/A
custom code	Zenodo&Github	https://doi.org/10.5281/zenodo.6037829
Other		
DLP 4500 LightCrafter unit	Texas Instruments	Cat # 296–36420-ND

## Highlights

- Rapid optogenetic Bcd control is combined with measurement of target gene dynamics
- *hb* and *gt* transcription responds in around 5 min to changes in nuclear Bcd
- Acute changes in Bcd concentration drive delayed repression of *Kr*
- *kni* transcription is initiated rapidly upon depletion of nuclear Bcd
