# Supplementary material for: Optogenetic control of the Bicoid morphogen reveals fast and slow modes of gap gene regulation
Source: Cell Rep. Author manuscript; Available in PMC 2022 Apr 20. (PMC9019726; doi:10.1016/j.celrep.2022.110543)
Supplement: 1 [file NIHMS1791913-supplement-1.pdf]

Cell Reports, Volume *38*

Supplemental information

Optogenetic control of the Bicoid morphogen reveals fast and slow modes of gap gene regulation

Anand P. Singh, Ping Wu, Sergey Ryabichko, João Raimundo, Michael Swan, Eric Wieschaus, Thomas Gregor, and Jared E. Toettcher

Supplementary Figures

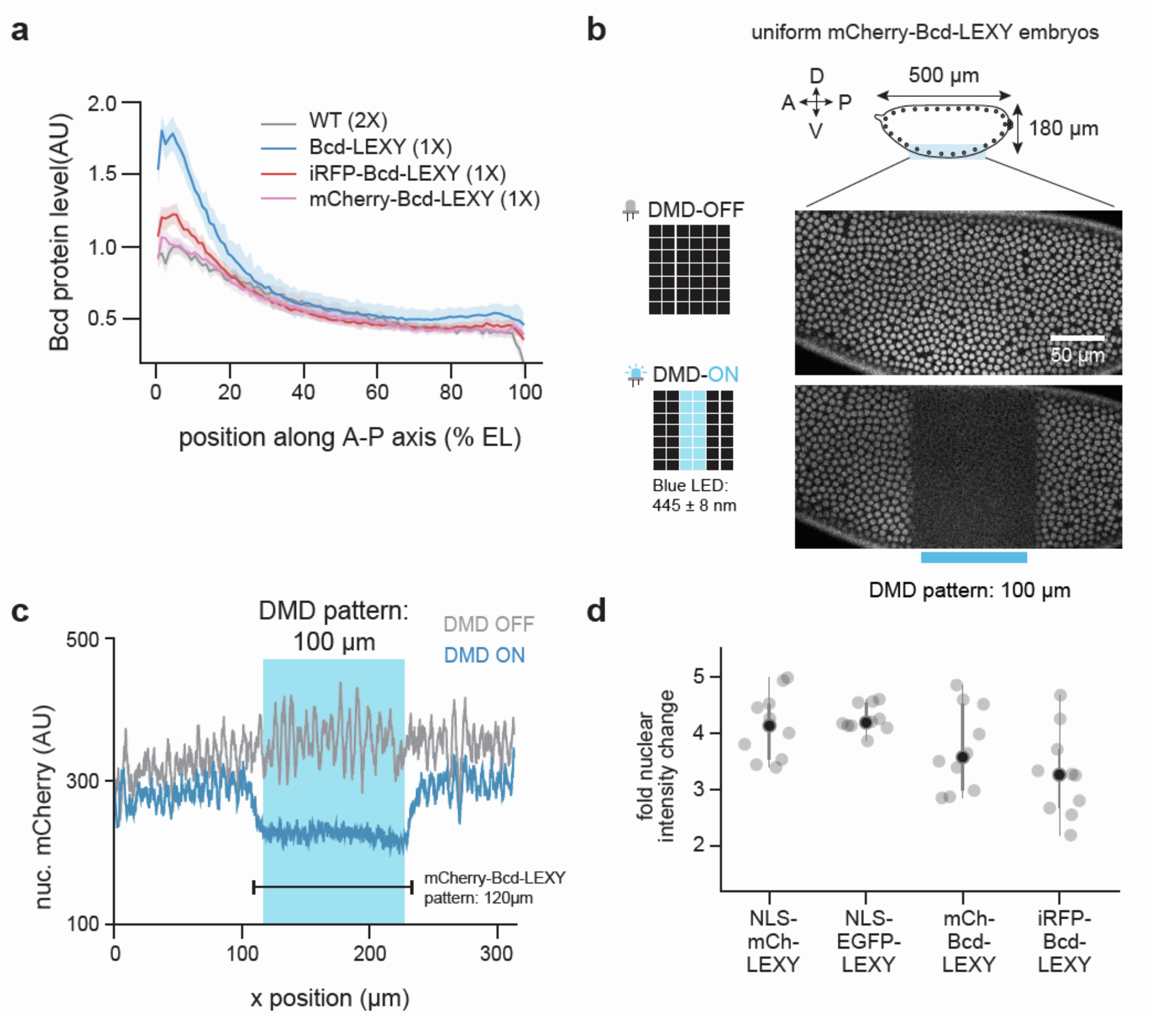

Figure S1. Characterization of Bcd-LEXY expression and translocation spatial precision and dynamics in different constructs. (**a**) Immunofluorescence for Bcd protein for three Bcd-LEXY constructs, compared to WT embryos. Bcd levels are quantified as a function of position and in the genetic background of a single copy of gradient αTub67C>Bcd-LEXY expressed in bcd^{E1} homozygous embryos. Error bars show SEM, n = 15 for WT, n = 30 for the other three genotypes. (**b-c**) Spatial precision of mCherry-Bcd-LEXY import and export. Representative images are shown in **b**, where the top panel shows the unilluminated state and bottom panel has a central stripe DMD pattern of 100 µm wide. As quantification of the pattern shown in **c**, the actual pattern on the embryo is 120µm wide, which is ~10-12µm wider (about 1-2 cell width) than the DMD pattern on both sides. (**d**) Nuclear fluorescence fold changes between dark and light conditions for different LEXY constructs. Fold change is quantified by division of background subtracted nuclear fluorescent intensity in dark condition by light condition. n = 10 for each LEXY constructs. *Related to Figure 2.*

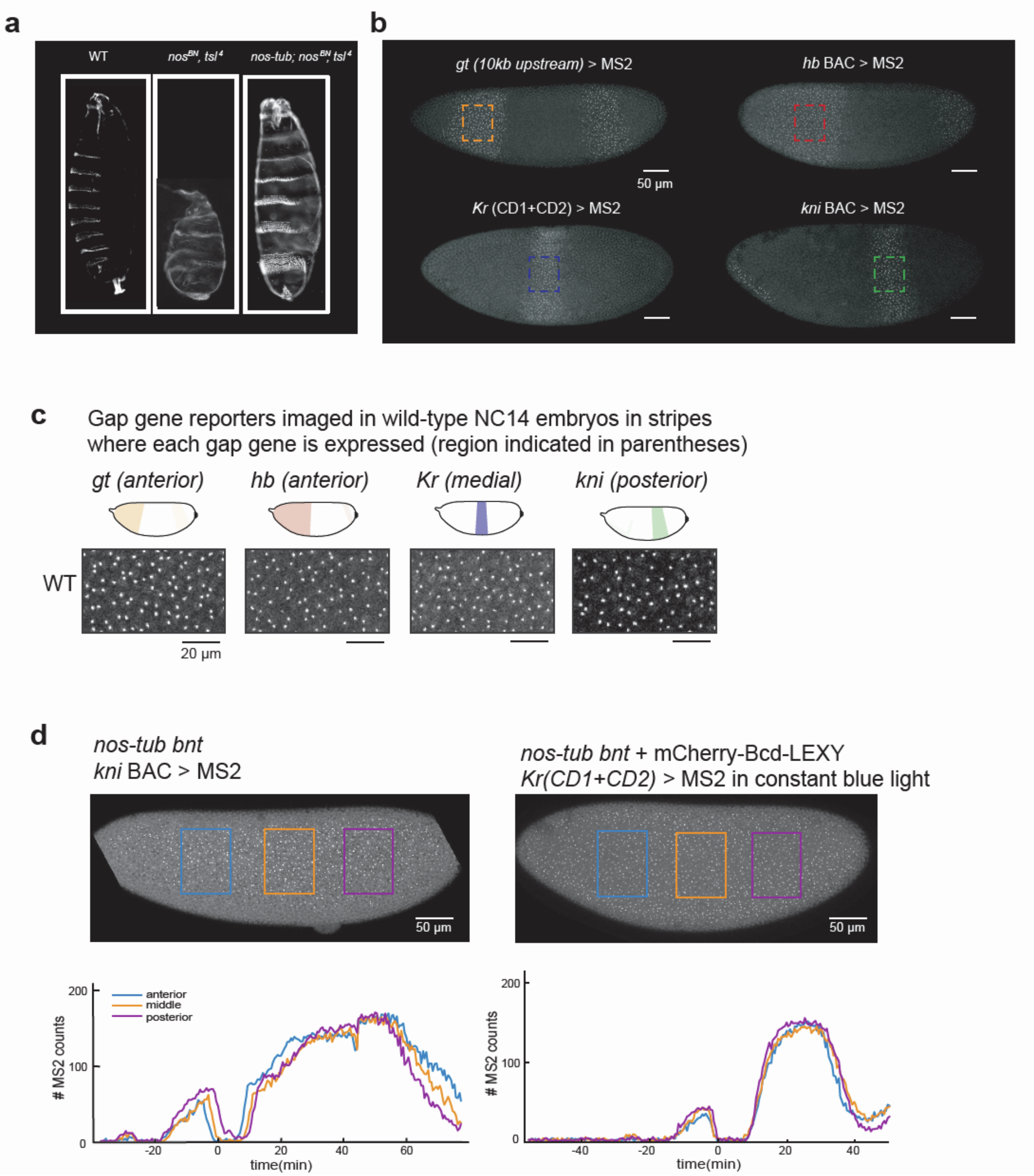

Figure S2. Phenotypic and genotypic characterization in WT, *nt* and *bnt* embryos. (**a**) Larval cuticles of *nos*^{BN}, *tsl*^{4} double mutant embryos and *nos*^{BN}, *tsl*^{4} double mutants with low uniform Nanos expression (*nos-tub*). *nos*^{BN}, *tsl*^{4} double mutant larva formed only 1-2 abdominal segments due to high Hb level at posterior part, whereas a low uniform expression of Nanos restores abdominal segments by repressing Hb translationally. (**b**) Characterization of MS2 reporters in WT embryos via MCP/MS2 imaging, which faithfully recapitulate endogenous gap gene expression pattern. (**c**) Cropped images from (**b**), 43.9 µm × 73.5 µm representative regions where four gap genes are expressed correspondingly, captured around 25-35min into NC14. (**d**) Time-lapse quantification of MS2 reporters in anterior, middle and posterior regions. By taking rectangles of the same size (43.9 µm × 73.5 µm) at anterior, middle and posterior

regions respectively, and quantifying the number of active transcription foci in each rectangle. Left panel shows *kni* BAC > MS2 in *nos-tub bnt* embryos, and the slight offset is due to division wave initiated from the anterior side. Right panel shows *Kr* (CD1 + CD2) > MS2 in *nos-tub bnt* + mCherry-Bcd-LEXY embryos. *Related to Figure 3.*

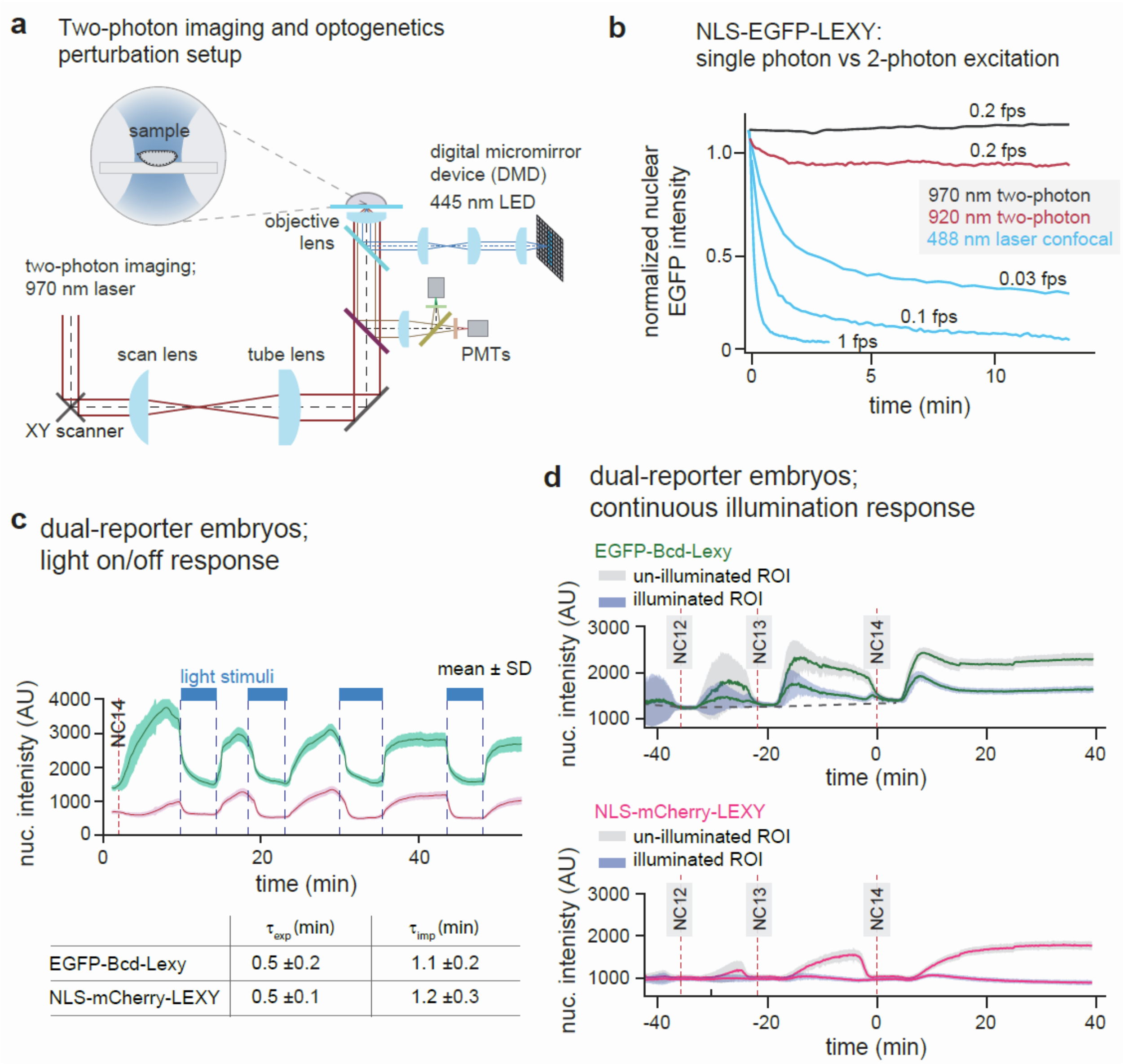

	τ_{exp} (min)	τ_{imp} (min)
EGFP-Bcd-Lexy	0.5 ±0.2	1.1 ±0.2
NLS-mCherry-LEXY	0.5 ±0.1	1.2 ±0.3

Figure S3. Two-photon setup for dual-color high-resolution imaging and optogenetic perturbation. (**a**) Microscope schematic of a two-photon laser confocal for live imaging and the DMD unit for optogenetics perturbations. (**b**) Comparison of single-photon 488 nm laser excitation and two-photon imaging of NLS-EGFP-LEXY (**Movie S1**). Whereas single-photon 488 nm laser and two-photon 920 nm laser still trigger activation of Aslov2 domain, thus resulting in nuclear export of NLS-EGFP-LEXY, two-photon 970 nm laser has no detectable effect on NLS-EGFP-LEXY. (**c**) Fast and reversible control of NLS-mCherry-LEXY and EGFP-Bcd-LEXY import and export in pulsatile cycles in NC14. Both constructs were co-expressed in the same embryo. Corresponding protein nuclear export & import rate time constant values are tabulated below. (**d**) Quantification of EGFP-Bcd-LEXY and NLS-mCherry-LEXY during NC11-14 in constant dark and light conditions. In the dark condition, NLS-mCherry-LEXY enters nuclei gradually after each division in contrast to EGFP-Bcd-LEXY (**Movie S1**). Furthermore, even in continuous blue light exposure, EGFP-Bcd-LEXY enriches in nuclei transiently right after division. This may be due to that Bcd tends to stick to DNA and right after nuclear envelope reforms, Bcd-LEXY might be brought into nuclei with DNA, which would potentially be faster than regular

nuclear import mediated by NLS (see **Movie S1**). For **c-d** shaded regions show standard deviation across > 180 nuclei in the same embryo. *Related to Figure 4.*

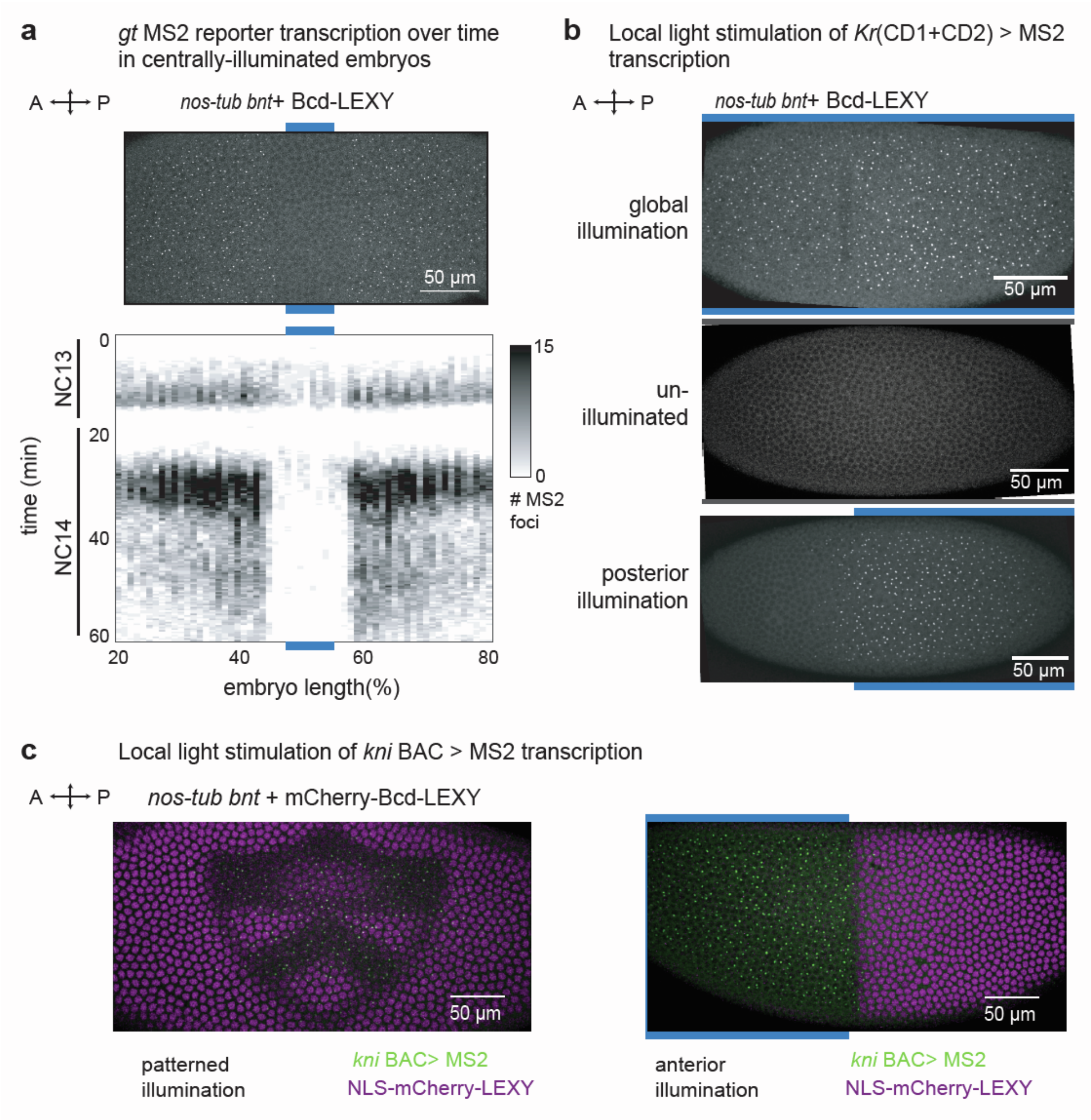

Figure S4. Spatial control of transcription response via optogenetic manipulation. (**a**) Time-lapse spatial pattern of *gt* > MS2 reporter in Bcd-LEXY embryos illuminated with central stripe pattern (40x150 μm^2). Light is on from the time 0, and the embryo showed relatively sharp and stable boundaries induced by spatial light pattern across time, especially in NC 14. (**b**) Different spatial expression patterns of *Kr* (CD1+CD2) > MS2 induced by different light inputs in iRFP-Bcd-LEXY embryos. Top panel: the whole embryo was exposed in blue light, and *Kr* transcription showed up across the embryo; middle panel: whole embryo was kept in dark, which showed no *Kr* transcription foci; bottom panel: posterior half of the embryo was exposed in light, and *Kr* transcription foci only turned on where was stimulated. (**c**) Precise control of both NLS-mCherry-LEXY translocation (magenta) and transcription *kni* BAC > MS2 (green) in mCherry-Bcd-LEXY embryos with illumination patterns of Princeton University logo (left) and anterior half(right) created by the DMD unit. *Related to Figure 4-6.*

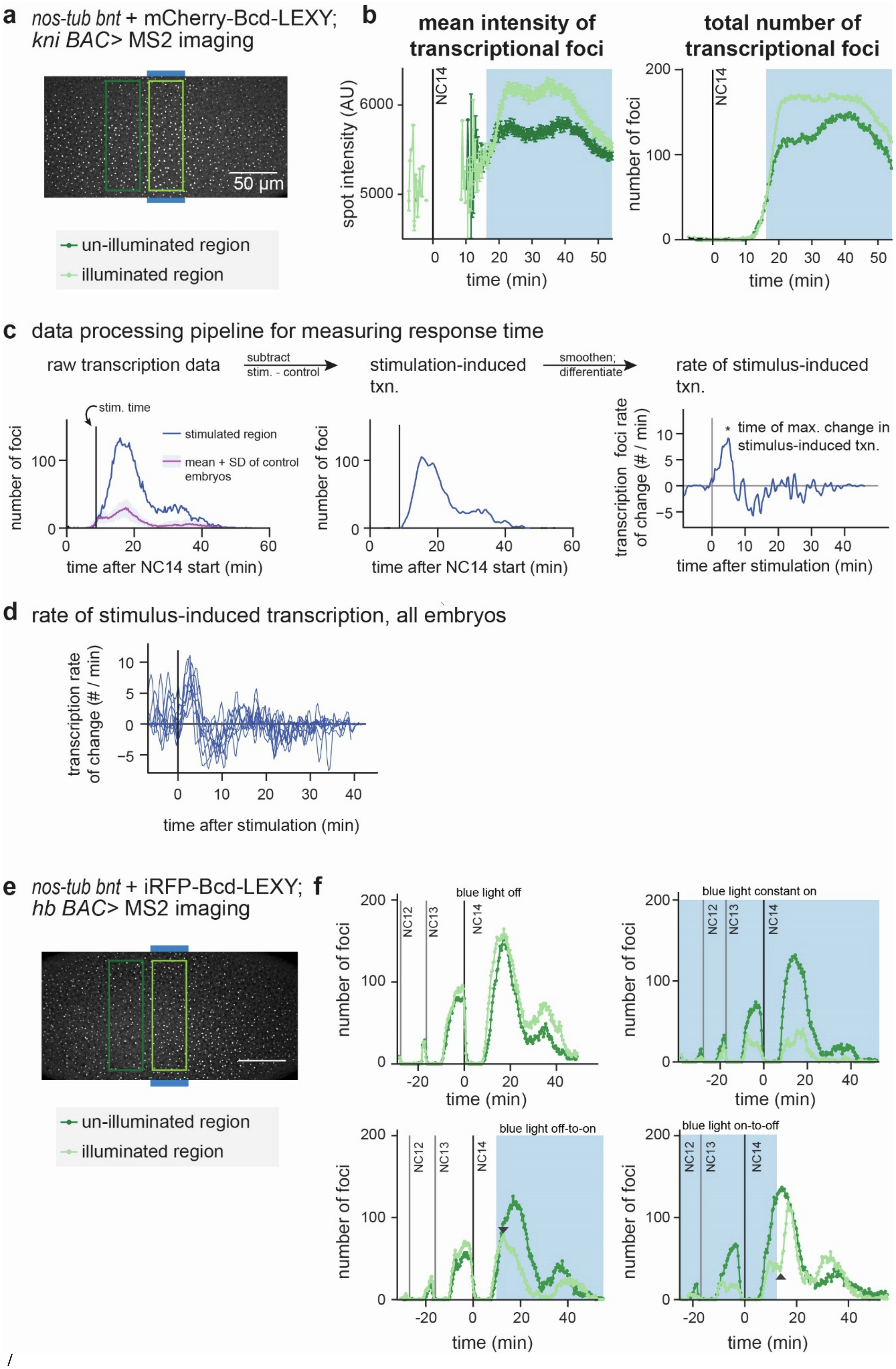

a
nos-tub bnt + mCherry-Bcd-LEXY; kni BAC> MS2 imaging
50 µm
un-illuminated region
illuminated region
b
mean intensity of transcriptional foci
spot intensity (AU)
NC14
time (min)
total number of transcriptional foci
number of foci
time (min)
c
data processing pipeline for measuring response time
raw transcription data
subtract stim. - control
stimulation-induced txn.
smoothen; differentiate
rate of stimulus-induced txn.
stim. time
stimulated region
mean + SD of control embryos
number of foci
time after NC14 start (min)
transcription foci rate of change (# / min)
* time of max. change in stimulus-induced txn.
time after stimulation (min)
d
rate of stimulus-induced transcription, all embryos
transcription rate of change (# / min)
time after stimulation (min)
e
nos-tub bnt + iRFP-Bcd-LEXY; hb BAC> MS2 imaging
un-illuminated region
illuminated region
f
blue light off
blue light constant on
blue light off-to-on
blue light on-to-off
NC12
NC13
NC14
number of foci
time (min)

/

Figure S5. Single embryo analysis of transcriptional dynamics of representative gap genes in different light stimulation conditions. (**a**) Representative image of *kni* BAC > MS2 in mCherry-Bcd-LEXY embryo at ~22 mins into nuclear cycle (NC)14, where the dark green box shows the un-illuminated region and the light green box shows illuminated region. (**b**) Time-lapse quantification of (**a**) Left panel: mean intensity of transcriptional foci across time, with error bar representing standard error of the mean; Right panel: total number of transcriptional foci. (**c**) Schematic of measuring stimulus response time from single embryo transcription time series data. A representative single embryo example of *hb* BAC> MS2 transcription foci count time series for on-to-off light stimulation condition. Magenta color plot shows mean foci count for 8 embryos in control light condition (continuous blue light stimulation). To determine light induced transcription, control condition mean transcription foci count data is subtracted from individual on-to-off data. Subsequently, derivative plots are generated followed by smoothening to extract time of maximum amplitude post stimulation (vertical bar representing time of stimulation is set to zero on the x-axis). The extracted time is referred to as response time post stimulation. (**d**) An example figure shows response time estimate plots for multiple embryos after the same operation from (**c**). (**e**) An example image of *hb* BAC > MS2 in iRFP-Bcd-LEXY embryo at ~15 mins into NC14. (**f**) Corresponding single embryo time-lapse quantification of transcription foci count with different optogenetic stimuli. Time 0 indicates the start of NC 14. Dark green curves represent un-illuminated region in each embryo, and light green curves represent central regions treated with different optogenetic stimuli, ranging from constant dark (top left), constant light (top right), dark-to-light switch in early NC14 (bottom left) and light-to-dark switch in early NC14 (bottom right). The black triangle indicates divergence of transcription foci count post blue light treatment compared to control un-stimulated ROI (bottom panel). *Related to Figures 4-6.*

.

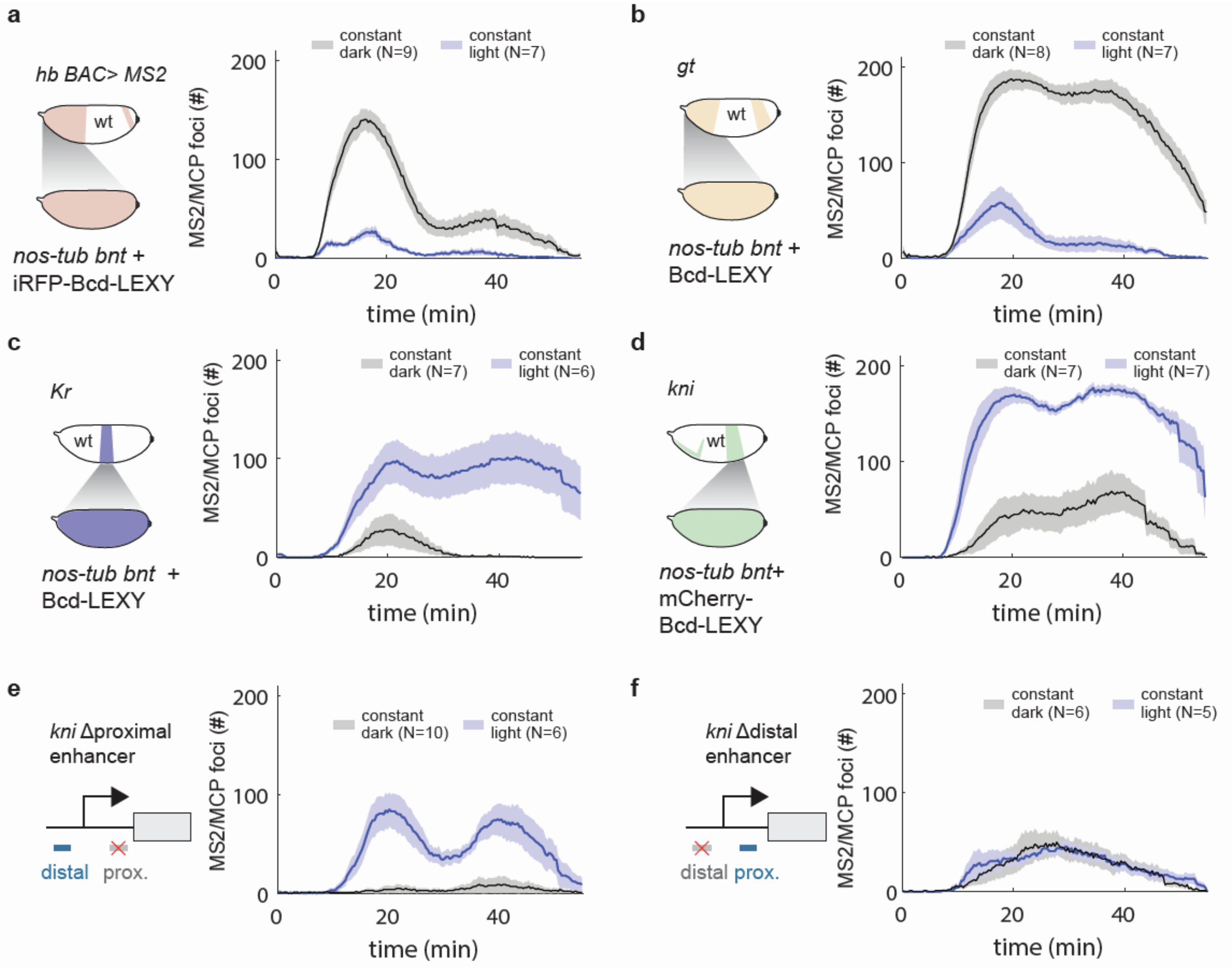

Figure S6. Live transcription measurement of gap genes in constant dark and light conditions. (**a-b**) Quantification of *hb* and gt transcriptional activity in constant dark and light conditions via MCP/MS2.*hb* and *gt* show high expression in dark and low expression in constant blue light, indicating that Bcd acts as a positive regulator for *hb* and *gt* (**Movie S2**). (**c-d**) Quantification of *Kr* and *kni* transcriptional activity in constant dark and light conditions via MCP/MS2 (**Movie S2**). In contrast to *hb* and *gt*, *Kr* and *kni* MS2 show higher expression constant light than dark condition, indicating that Bcd may act as a negative regular for *Kr* and *gt*. (**e-f**) *kni* Δproximal and *kni* Δdistal enhancer driven MS2 in constant dark and light conditions (**Movie S3**). *kni* Δproximal enhancer has much higher transcription activity in light compared to dark, similar to full *kni* BAC > MS2 in **d**, whereas *kni* Δdistal enhancer barely has any changes. Thus, *kni* distal enhancer is mainly responsible for the change observed via optogenetic control. For **a-f** shaded regions show standard error of the mean, and the number of embryos tested is indicated in the legend of each plot. *Related to Figures 4-6*.

Supplementary Tables

Table S1. Hatch rates and cephalic furrow positions in gradient optogenetic Bcd-LEXY variants. Related to Figure 2.

**Genotype**	**Hatch rate, dark**	**Hatch rate, light**	**Cephalic furrow position, dark** Mean ±SD(%EL)	**Cephalic furrow position, light** Mean ± SD(%EL)
WT	92.3% (192/209)	87.9% (182/207)	33.2±1.7 (N=8)	33.6±1.4 (N=10)
Bcd-LEXY, bcd^{E1}/bcd^{E1}	69.9% (269/385)	0% (0/49)	37.5±1.4 (N=12)	26.1±1.9 (N=11)
iRFP-Bcd-LEXY, bcd^{E1}/bcd^{E1}	42.0% (131/312)	0% (0/25)	27.1±2.4 (N=16)	Not formed (N=7)
mCherry-Bcd-LEXY, bcd^{E1}/bcd^{E1}	0% (0/78)	0% (0/22)	Not formed (N=8)	Not formed (N=5)

Table S2. Response time for each gap gene after optogenetic stimulation. Related to Figures 4-6.

**MS2 reporter**	**Mean response time post light perturbation mean ± SEM**	
	**Light On- Off transition**	**Light Off- On**
hb	1.7 ± 0.9 (11/12 embryos analyzed); **Figure 4d**	3.3 ± 1.1 (9/10); **Figure 4e**
gt	5.9 ± 1.6 (6/9); **Figure 4g**	9 ± 2.7 (5/6) **Figure 4h**
Kr	21.7 ± 1.8 (7/8); **Figure 5b**	NA (no change); **Figure 5c**
kni	3.5 ± 1.7 (7/9); **Figure 6b**	2.9 ± 0.9 (7/7); **Figure 6c**

Table S3. Live protein and mRNA reporter lines and imaging parameters. Related to Figures 3-6.

Reporter line	Imaging conditions	DMD light conditions
αTub67C> NLS-EGFP-LEXY (**Figure 2h**)	Custom built two-photon microscope<br>laser-970 nm<br>laser power- 10 mW before objective<br>z-slice step= 1.1 μm<br>total z-slices= 2<br>single time frame=5 sec<br>pixel size=300 nm Image resolution =1024x512 pixels	DMD blue light wavelength= 445 nm<br>Pulse duration=40 ms<br>Pulse duty cycle= 100 ms<br>Number of pulses= 5<br>Blue light power 100 $\mu W/cm^2$
αTub67C> NLS-mCherry-LEXY (**Figure 2h**)	same as above	same as above
αTub67C> EGFP-uBcd-LEXY (**Figure S3c-d**)	same as above	same as above
αTub67C> iRFP-uBcd-LEXY (**Figure 2h**)	**Nikon A1R** laser-scanning confocal<br>640 nm 20X air objective<br>laser power - 10%<br>PMT HV 120, PMT offset 8<br>time interval = 5 sec<br>pixel size=300 nm<br>image resolution =1024x512 pixels	DMD blue light wavelength= 445 nm<br>Blue light power 25-30 $\mu W/cm^2$<br>constant
αTub67C> mCherry-uBcd-LEXY (**Figure 2h**)	561 nm<br>rest same as above	DMD blue light wavelength= 445 nm<br>Blue light power 26 $\mu W/cm^2$<br>constant
gt (**Figure 4**)	custom built two-photon microscope<br>laser-970 nm<br>laser power- 10 mW before objective<br>z-slice step= 1.1 μm<br>total z-slices= 8<br>single time frame=20 sec<br>pixel size=300 nm<br>image resolution = 1024x512 pixels	DMD blue light wavelength= 445 nm<br>Pulse duration=40 ms<br>Pulse duty cycle= 100 ms<br>Number of pulses= 5<br>Blue light power 100 $\mu W/cm^2$
hb BAC (**Figure 4**)	same as above	same as above
Kr (**Figure 5**)	same as above	same as above
kni BAC (**Figure 6**)	same as above	same as above
kni BAC ΔDist (**Figure 6**)	same as above	same as above
kni BAC ΔProx (**Figure 6**)	same as above	same as above
